# Phosphorylation-Driven Epichaperome Assembly: A Critical Regulator of Cellular Adaptability and Proliferation

**DOI:** 10.21203/rs.3.rs-4114038/v1

**Published:** 2024-04-03

**Authors:** Seth W. McNutt, Tanaya Roychowdhury, Chiranjeevi Pasala, Hieu T. Nguyen, Daniel T. Thornton, Sahil Sharma, Luke Botticelli, Chander S. Digwal, Suhasini Joshi, Nan Yang, Palak Panchal, Souparna Chakrabarty, Sadik Bay, Vladimir Markov, Charlene Kwong, Jeanine Lisanti, Sun Young Chung, Stephen D. Ginsberg, Pengrong Yan, Elisa DeStanchina, Adriana Corben, Shanu Modi, Mary Alpaugh, Giorgio Colombo, Hediye Erdjument-Bromage, Thomas A. Neubert, Robert J. Chalkley, Peter R. Baker, Alma L. Burlingame, Anna Rodina, Gabriela Chiosis, Feixia Chu

**Affiliations:** 1. Department of Molecular, Cellular & Biomedical Sciences, University of New Hampshire, Durham, NH 03824, USA.; 2. Chemical Biology Program, Memorial Sloan Kettering Cancer Center, New York, NY 10065, USA.; 3. Antitumor Assessment Core Facility, Memorial Sloan Kettering Cancer Center, New York, NY 10065, USA.; 4. Departments of Psychiatry, Neuroscience & Physiology & the NYU Neuroscience Institute, NYU Grossman School of Medicine, New York, NY, 10016, USA.; 5. Center for Dementia Research, Nathan Kline Institute, Orangeburg, NY, 10962, USA.; 6. Department of Pathology, Memorial Sloan Kettering Cancer Center, New York, NY, 10065, USA.; 7. Department of Medicine, Division of Solid Tumors, Memorial Sloan Kettering Cancer Center, New York, NY 10065, USA.; 8. Department of Chemistry, University of Pavia, via Taramelli 12, 27100 Pavia, Italy.; 9. Department of Neuroscience and Physiology and Neuroscience Institute, NYU Grossman School of Medicine, New York, NY, 10016, USA.; 10. Mass Spectrometry Facility, University of California, San Francisco, California 94143, USA.; 11. Hubbard Center for Genome Studies, University of New Hampshire, Durham, NH 03824, USA.; 12 co-first author, equally contributed to the work.; 13 These authors jointly supervised this work: Feixia Chu, Gabriela Chiosis.

## Abstract

The intricate protein-chaperone network is vital for cellular function. Recent discoveries have unveiled the existence of specialized chaperone complexes called epichaperomes, protein assemblies orchestrating the reconfiguration of protein-protein interaction networks, enhancing cellular adaptability and proliferation. This study delves into the structural and regulatory aspects of epichaperomes, with a particular emphasis on the significance of post-translational modifications in shaping their formation and function. A central finding of this investigation is the identification of specific PTMs on HSP90, particularly at residues Ser226 and Ser255 situated within an intrinsically disordered region, as critical determinants in epichaperome assembly. Our data demonstrate that the phosphorylation of these serine residues enhances HSP90’s interaction with other chaperones and co-chaperones, creating a microenvironment conducive to epichaperome formation. Furthermore, this study establishes a direct link between epichaperome function and cellular physiology, especially in contexts where robust proliferation and adaptive behavior are essential, such as cancer and stem cell maintenance. These findings not only provide mechanistic insights but also hold promise for the development of novel therapeutic strategies targeting chaperone complexes in diseases characterized by epichaperome dysregulation, bridging the gap between fundamental research and precision medicine.

## Introduction

Conventional wisdom, as crystallized in Beadle and Tatum’s 1941 paradigm of “one gene–one enzyme–one function,” has traditionally delineated targets as outcomes of protein expression changes or point mutations within proteins. However, it is increasingly apparent that protein dysfunctions in the context of many disorders, including cancer, neurodegenerative disorders, among others, are predominantly shaped by changes in interaction strengths and cellular mislocalization. These factors, in turn, can be modulated by variations in post-translational modifications (PTMs), stabilization of disease-associated protein conformations, and other protein-modifying mechanisms^1,2^. Within this complex context, Heat Shock Protein 90 (HSP90) emerges as a compelling exemplar, transcending the boundaries of conventional understanding^3^.

Positioned as a versatile chaperone, often referred to as the guardian of the proteome, HSP90 assumes a pivotal task in the realm of maintaining cellular equilibrium by facilitating protein folding, stabilization, and degradation^4^. Under the canonical folding paradigm, HSP90 functions as a homodimer. Each protomer is composed of an N-terminal domain (NTD), a middle domain (MD), and a C-terminal dimerization domain (CTD)^4,5^. The NTD contains a nucleotide binding pocket, where ATP binding and hydrolysis takes place^6^. The chaperone cycle of HSP90 is coupled to a series of dynamic conformational changes accompanying its ATPase cycle. Beginning with NTD/MD and MD/CTD interdomain rotations and cross-monomer dimerization^7^, HSP90 transitions from open to closed conformational states, while folding client proteins^8,9^. HSP70 and HOP (HSP70-HSP90 organizing protein) bring client proteins to HSP90 and form the loading complex^10^. Other co-chaperones participate at different stages of the HSP90 chaperone cycle and regulate its conformational changes along the chaperone and ATPase cycle^4^. Co-chaperones may have different preferences for client proteins, fine-tuning subcellular pools of HSP90 to mitigate stressors and maintain proteostasis^11^. These assemblies are further shaped by PTMs in HSP90, co-chaperones and client proteins^12^. Overall, the highly orchestrated interactions among these proteins – both chaperones and clients – are transient in the chaperone cycle under physiological conditions.

While this classical understanding portrays HSP90 as a dimeric entity that interacts dynamically with co-chaperones and client proteins, research has uncovered a spectrum of multimeric HSP90 forms, each sculpted by the cellular milieu and the presence of stress-inducing factors^3^. These multimers, whether homo-oligomeric or hetero-oligomeric, expand HSP90’s functional repertoire, blurring the boundaries between traditional chaperone functions and newfound roles as holdases or scaffold proteins. In disease contexts, such as cancer and neurodegenerative disorders, HSP90’s conformational adaptability gives rise to epichaperomes—distinctive hetero-oligomeric formations of tightly bound chaperone, co-chaperones and other factors^13–15^. This phenomenon goes beyond mere biochemical curiosity; it represents a fundamental mechanism by which cells respond to stressors, whether of genetic, proteotoxic or environmental nature^3,16–18^. Unlike chaperones which help proteins fold or assemble, epichaperomes exert a maladaptive influence, reshaping the assembly and connectivity of proteins pivotal for sustaining pathological traits. For example, in cancer, epichaperomes take on scaffolding functions not found in normal cells, altering the assembly and connectivity of proteins important for maintaining a malignant phenotype and enhancing their activity, which provides a survival advantage to cancer cells and tumor-supporting cells^13,19^. In Alzheimer’s disease epichaperomes rewire the connectivity of, and thus negatively impact, proteins integral for synaptic plasticity, brain energetics and immune response^15^.

The revelation of HSP90’s maladaptive multimeric epichaperomes has also profound implications for therapeutic interventions, including in the treatment of diverse disease states including cancers and of neurodegenerative disorders. Rather than a blanket inhibition of all HSP90 pools, targeting specific pathologic conformations of HSP90 as found in epichaperomes while sparing normal HSP90 functions holds the promise of enhancing the safety as well as the immunostimulatory and anticancer effects of HSP90 inhibitors^3^.

Despite these important mechanistic and therapeutic implications, key factors facilitating HSP90 incorporation in epichaperomes – namely the conformations that enable epichaperome formation and structural elements that support the enrichment of such conformation - remain unknown. In this study, we use a combination of chemical biology and unbiased mass spectrometry techniques to elucidate the conformation of HSP90 populated in epichaperomes and to characterize molecular factors that support and favor the enrichment of such conformation. Beyond structural revelations, our findings demonstrate how these factors directly influences cellular behaviors, particularly in contexts where robust proliferation and adaptation are crucial, such as cancer and stem cell maintenance. This direct link between epichaperome function and fundamental cellular processes has translational relevance for therapeutic development.

## RESULTS

### Pluripotent stem cells and cancer cells share epichaperomes

Epichaperomes nucleated through enhanced interactions between HSP90 and HSP70, namely the heat shock cognate 70 (HSC70) isoform, are a distinct feature of cancer cells^13,19^. Epichaperomes containing HSP90 are detected in iPSCs (induced pluripotent stem cells)^20^, in leukemia stem cells^21,22^ and in glioma cancer stem cells (CSCs)^23^. Hyperactivation of the transcription factor c-MYC required in generating iPSCs^24^, maintaining embryonic stem cells (ESCs)^25^ and CSCs^26^, is also a driving factor in epichaperome formation in tumors, irrespective of the tumor type^13,27^. Notably, these epichaperomes are all sensitive to and can be disrupted by small molecules such as PU-H71 (zelavespib) or PU-AD (icapamespib) that bind to HSP90^13,23,28^, suggesting that a similar composition, facilitated by a specific conformation of HSP90, may characterize epichaperomes in these distinct cellular contexts.

**Figure 1.**
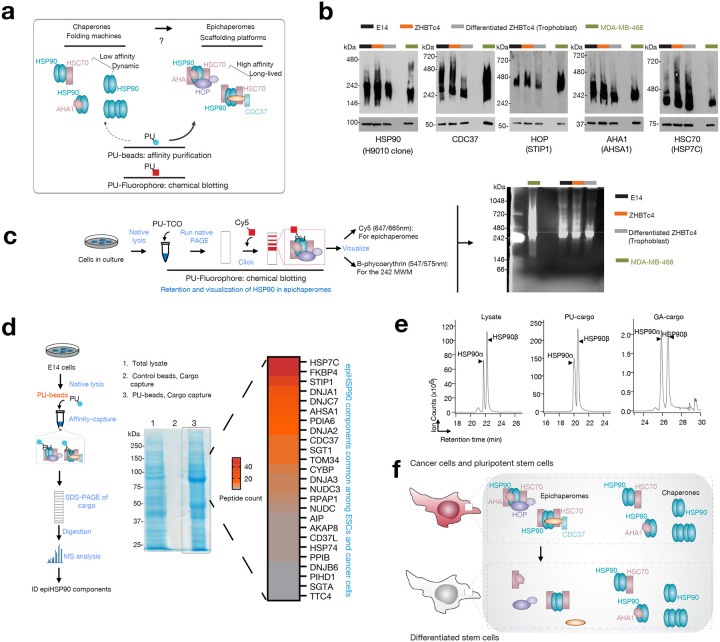
Embryonic stem cells and cancer cells share compositionally similar epichaperomes. **a** Schematic illustrating the biochemical and functional distinctions between epichaperomes, defined as long-lasting heterooligomeric assemblies composed of tightly associated chaperones and co-chaperones, and traditional chaperones. Unlike chaperones, which assist in protein folding or assembly, epichaperomes sequester proteins, reshaping protein-protein interactions, and consequently altering cellular phenotypes. The schematic also outlines key principles for the use of PU-probes in epichaperome analysis. **b** Detection of epichaperome components (chaperones and co-chaperones) through SDS-PAGE (bottom, total protein levels) and native-PAGE (top), followed by immunoblotting. See also Supplementary Fig. 1. **c** Visualization of HSP90 in epichaperomes using the PU-TCO click probe. See also Supplementary Fig. 2. Gel images are representative of three independent experiments. **d** Epichaperome constituent chaperones and co-chaperones identified through mass spectrometry analyses of PU-beads cargo. Representative data of two independent experiments. See Supplementary Fig. 3 for the GA-cargo. **e** Illustration of an isobaric, discriminant peptide pair from ESC lysate samples and HSP90 captured by PU- and GA-beads. Representative data of two independent experiments. **f** Schematic summary. Both cancer cells and pluripotent stem cells harbor epichaperomes. These epichaperomes undergo disassembly during differentiation processes. Source data are provided in Supplementary Data 1 and in Source data file.

To test this hypothesis, we initially explored the composition of epichaperomes in selected cellular models, encompassing pluripotent stem cells and cancer cells. For pluripotent stem cells, we examined two mouse embryonic stem cell lines (E14 and ZHBTc4) and a human induced pluripotent cell line (hiPSC). Additionally, two cancer cell lines, well-characterized in terms of epichaperome composition and function, were chosen as representative epichaperome-positive (MDA-MB-468) and -negative/low (ASPC1) cancer cells (Fig. 1a–f and **Supplementary Figs. 1,2**).

In contrast to folding chaperone complexes, which are inherently dynamic and short-lived^6^, epichaperomes represent long-lasting heterooligomeric assemblies composed of tightly associated chaperones, co-chaperones, and various other factors. HSP90 is a major component found within epichaperomes along with other chaperones, co-chaperones, and scaffolding proteins like HSP70 (especially HSC70), CDC37, AHA1, and HOP^13^. Consequently, when we analyzed cell homogenates containing epichaperomes using native PAGE followed by immunoblotting with antibodies specific to epichaperome constituent chaperones and co-chaperones, we observed a range of high-molecular-weight species, both distinct and indistinct, in addition to the primary band(s) characteristic of chaperones. This observation held true for both pluripotent stem cells and cancer cells (Fig. 1b, **Supplementary Fig. 1a-d** and refs.^13,19,20^). Notably, HSP90 immunoblotting revealed the presence of species comprising HSP90 in epichaperome assemblies in cancer cells and pluripotent stem cells, in addition to the prominent 242 kDa band, which is a characteristic of non-transformed cells^13,19,29^.

Epichaperomes undergo disassembly during iPSC differentiation^20^ or when cancer cells are treated with PU-H71 or PU-AD^15,23,28,30^. Therefore, next we induced the differentiation of the pluripotent stem cells under investigation. In the ZHBTc4 cell line, Oct4 expression is controlled by a Tet (tetracycline)-*off oct4* regulatory system^31^. Down-regulation of Oct4 in ZHBTc4 cells has been reported to induce trophoblast differentiation, which is characterized by changes in cell morphology, specifically, cells flattening into epithelial-like cells, and is associated with slower growth^32^. Mouse embryonic E14 stem cells undergo spontaneous differentiation into embryoid bodies when cultured in suspension without antidifferentiation factors such as leukemia inhibitory factor^33^ and induced pluripotent stem cells differentiate into mature dopaminergic neurons using a floor-plate based differentiation protocol^34^. We confirmed that differentiation of these pluripotent stem cells was correlated with the disassembly of epichaperomes, as observed through native PAGE immunoblotting. This disassembly is evident by a reduction in high-molecular-weight chaperone species on native PAGE observed when immunoblotting for epichaperome constituent chaperones (see HSP90α/β, HOP, HSC70, CDC37, AHA1, HSP110 in Fig. 1b and **Supplementary Fig. 1**), with minimal changes observed in total chaperone levels on SDS PAGE. Notably, for HSP90, a decrease in bands other than those in the ~242 kDa range was observed upon differentiation, supportive of epichaperome disassembly (see HSP90 immunoblotting).

PU-H71 serves as an epichaperome probe that, in contrast to the tested antibodies which indiscriminately detect epichaperomes and other HSP90 pools, exhibits a preference for HSP90 when it is integrated into epichaperomes^13^. Labeled derivatives of PU-H71 can, therefore, be employed to detect HSP90 within epichaperomes, distinguishing it from other HSP90 pools (as illustrated in Fig. 1c and **Supplementary Fig. 2a-c**). To achieve this, we generated lysates from ZHBTc4, E14 cells, and MDA-MB-468 cells under conditions that preserve native protein assemblies. Subsequently, we labeled these homogenates with a clickable PU-probe (PU-TCO, ref. 19). After running these labeled samples on native PAGE gels, we conjugated the PU-probe with a Cy5 dye and visualized epichaperomes, confirming the presence of epichaperomes in both the ESCs and the cancer cells. These epichaperomes were characterized by multimers observed at and above ~300 kDa (Fig. 1c). Moreover, the labeling of epichaperomes by the PU-probe decreased upon ESC differentiation, supportive of epichaperome disassembly (Fig. 1c and **Supplementary Fig. 2b**).

Additionally, we conducted labeling experiments using live E14 ESCs, instead of homogenates, employing a PU-CW800 probe (a derivative of PU-H71 conjugated with an 800 nm near-infrared dye) or a control derivative (an inactive PU-derivative that does not interact with epichaperomes) (see **Supplementary Note 1**). The most responsive target of the PU-probes, but not the control probe, was an HSP90 assembly of approximately 300 kDa, thus above the major 242 kDa band preferred by the anti-HSP90 antibody. This species was detected on Native-PAGE in PU-probe treated cell lysates but not in control treated cell lysates (**Supplementary Fig. 2c**).

In summary, the predominant HSP90 band characteristic of epichaperomes is a 300 kDa assembly, distinctly differing from the typical ~242 kDa band observed in non-transformed cells^13, 19, 32^ when analyzed on native PAGE gels. Mass spectrometric (MS) analysis of the ~300 kDa assembly confirmed the presence of HSP90 and HSC70 as the primary protein components of this multimeric complex (**Supplementary Data 1**, 300 kDa LC-MS). This finding aligns with the composition of core epichaperome complexes previously reported in cancer cells^13^. Consequently, these findings combined confirm that both cancer cells and pluripotent stem cells share HSP90 and HSC70 as integral constituents of their core epichaperomes.

To gain further insights into epichaperome assemblies, we employed resin-based affinity purification experiments. Specifically, we utilized resins with immobilized PU-H71, referred to as PU-beads, and an inert control molecule on control beads, following established procedures^13^ (Fig. 1d). As an additional control, we employed a resin containing immobilized geldanamycin (GA), known for its ability to bind and isolate predominantly un-complexed HSP90 (GA-beads, **Supplementary Fig. 3** and ref.^35^). Subsequently, we subjected the protein cargo isolated by these probes to unbiased MS analysis. To precisely determine the protein components of the cargo, we conducted in-gel digestion of the entire gel lanes and employed liquid chromatography/mass spectrometry (LC-MS/MS) in conjunction with the semi-quantitative spectra-counting method^36,37^ for the identification and quantification of cargo proteins (**Supplementary Data 1**).

We observed that the cargo isolated by PU-beads from ESCs contained 26 of the 42 major chaperone and co-chaperones identified prior in cancer cells^13^ as being epichaperome components (Fig. 1d). The interaction between PU-beads and epichaperomes was specific towards PU-H71, because control resins did not purify noticeable protein complexes. Similarly, GA-beads precipitated HSP90 but few co-purifying proteins and epichaperome components (**Supplementary Fig. 3, Supplementary Data 1**) consistent with previous results that GA isolates largely an un-complexed HSP90^38^.

In mammalian cells, HSP90 exists in two paralogs, HSP90α and HSP90β^39^, both of which have been reported to play roles in epichaperome formation in cancer cells^13^. To assess the isoform composition of HSP90 within epichaperomes, we exploited the subtle difference between one pair of isobaric peptides, namely 88Thr-Lys100 in HSP90α and 83Thr-Lys95 in HSP90β, where a single amino acid distinguishes them (Ile in HSP90α and Leu in HSP90β) (**Supplementary Fig. 4a**). The assignment of HSP90 isoforms relied on co-eluting peptides obtained from the isobaric peptide present in purified HSP90β (**Supplementary Fig. 4b,c**). Extracted ion chromatograms of the peptide mass revealed an approximate ~1.5 β/α ratio in the ESC lysate and the cargo isolated by PU-beads (Fig. 1e), while the GA-beads cargo exhibited a ~1.0 β/α ratio. Similar findings were obtained through spectra counting, with the HSP90β/HSP90α ratio determined using spectral counting consistent with ratios obtained through MS intensity calculations (**Supplementary Data 1**: 708/540 = 1.31 for the PU-beads cargo; 219/235 = 0.93 for the GA-beads cargo). This validation of spectra counting as an effective semi-quantitative method supports the conclusion that epichaperomes isolated from ESCs exhibit a predominantly unbiased HSP90 paralog composition, akin to what has been reported for cancer cells^13^.

In summary, the wealth of complementary biochemical experiments presented here lends strong support to the idea that both cancer cells and pluripotent stem cells harbor epichaperomes that are compositionally similar. Notably, HSP90 and HSC70 emerge as major constituents of the core epichaperome structure, serving as a scaffold for recruiting various co-chaperones to create specific epichaperome assemblies. This shared architectural similarity between epichaperomes in ESCs and cancer cells underscores the existence of a common epichaperome-enabling HSP90 conformer that is enriched in both biological contexts.

### Epichaperome-enabling conformation of HSP90

MS identification of cross-linked residues that are in spatial proximity but not necessarily close in primary sequence, provides valuable distance restraints that can be employed for computational modeling of proteins and protein complexes^40–42^. Therefore, to determine the conformation of HSP90 in epichaperomes, we used a chemical cross-linking and mass spectrometry (CX-MS) approach to identify and quantify cross-linked peptides of PU-H71-favored HSP90 pools.

**Figure 2.**
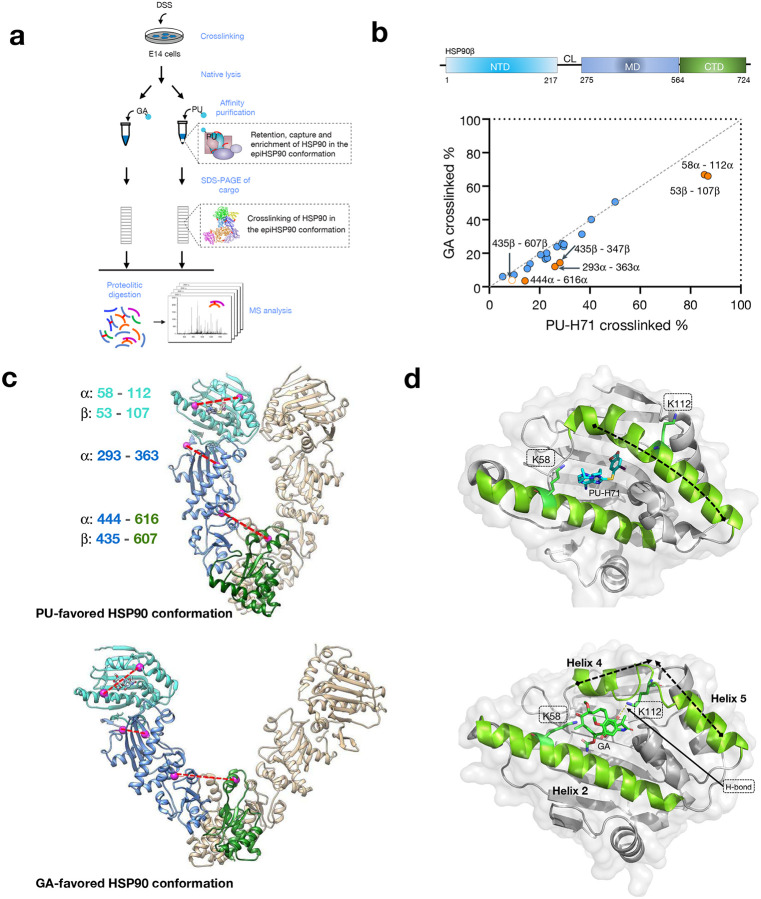
An enrichment of the closed-like conformation of HSP90 favors epichaperomes formation. **a** Experiment outline. **b** Plot comparing cross-linking propensity of Lys residues in HSP90 bound to PU-H71 or GA. Average cross-linking percentage of PU-H71 (x-axis) and GA (y-axis) bound HSP90 cross-linked pairs are shown. Blue circles represent pairs with similar cross-linking propensity (dotted line with a slope of 1). Orange points indicate outlier cross-linked peptides, with cross-linked Lys residues 8 amino acids away and the cross-linking percentage difference ≥ 1.5 standard deviation of replicates. Solid orange circles represent p ≤ 0.05, n = 3 replicate measurements. **c** Homology model illustrating the HSP90 dimer in the open conformation (template PDB: 2IOQ), favored by geldanamycin (GA), and the closed conformation (template PDB: 2CG9), favored by PU-H71. One HSP90 protomer is colored to indicate the N-terminal domain (NTD, light blue), the middle domain (MD, dark blue), and the C-terminal domain (CTD, green). Cross-linked residues are labeled by pink dots and connected by red dashed lines. **d** NTD structures of PU-H71 (top, PDB: 2FWZ) and GA (bottom, PDB: 1YET)-bound HSP90. Source data are provided as Supplementary Data 2.

To ensure the capture of the epichaperome-enabling conformation, we first cross-linked cellular lysates using the amine-reactive cross-linker DSS (disuccinimidyl suberate) prior to HSP90 capture on the PU-beads^13,35^ (Fig. 2a). Parallel experiments were conducted using GA-beads, corresponding to solid-support immobilized GA, as a control^13,35^. The identity of cross-linked HSP90 peptides purified by PU- or GA-beads pull-down can be found in **Supplementary Data 2**. Notably, the alpha carbon distances between all cross-linked residues, as identified with high confidence, fell below the maximal span of DSS (30 Å). This suggests that proteins retained their native states without significant conformational perturbations during the cross-linking process.

We calculated the cross-linking percentage for each pair of cross-linked PU- or GA-bound HSP90 residues. This calculation involved normalizing the MS ion intensity of cross-linked peptides by the sum of all cross-linked peptides and cross-linker-modified peptides containing the cross-linked residues. By doing so, we could mitigate the impact of variations in the reactivity of cross-linked residues, allowing us to primarily assess the influence of the distance between cross-linked residues and their local secondary structures^43^.

Most cross-linked pairs from both PU- and GA-bound samples exhibited similar cross-linking percentages, with data points evenly distributed around a trend line with a slope of 1 (dotted line, Fig. 2b). This observation suggests a broad similarity in secondary and tertiary structures between these HSP90 populations. However, clear differences emerged, revealing conformational distinctions between the PU- and GA-favored HSP90 subpopulations (highlighted by orange circles, Fig. 2b).

Notably, residues Lys58-Lys112 in HSP90α and Lys53-Lys107 in HSP90β, situated within the ligand-binding pocket, displayed a higher cross-linking percentage in PU-bound HSP90 populations compared to their GA-bound counterparts (Fig. 2b). This observation aligns with distinct pocket configurations preferred by each ligand, as previously observed through X-ray crystallography^44–48^. Specifically, crystal structures show the bulkier GA binds more superficially, causing helices 4 and 5 (Fig. 2d) to move away from the nucleotide binding site, thereby preventing full closure of the ATP lid. Moreover, the side-chain amino functional group of Lys112 forms a hydrogen bond with a benzoquinone oxygen of GA. This pocket configuration aligns with the reduced cross-linking activity of the lysine pair mentioned above. Conversely, PU-H71 binds deeply within the pocket. In this configuration, helices 4 and 5 are packed against helix 2 with Lys112 and Lys58 in HSP90α (or Lys107 and Lys53 in HSP90β) positioned more favorably for cross-linking. This arrangement of lysine residues is more likely to be found in the closed conformation of HSP90 (Fig. 2c), as proposed by crystallographic studies (PDB: 2CG9)^49^.

It is essential to reiterate that the cross-linking experiments were conducted to ‘lock’ HSP90 conformations with covalent bonds before resin-based affinity purification experiments using the PU- or GA-beads. Consequently, the X-ray structures of PU- or GA-bound HSP90 NTD closely reflect a preferred pocket configuration that each ligand may capture in the cell, and in this case, for PU-H71, it is indicative of the pocket configuration of HSP90 in the epichaperomes.

Furthermore, differences in HSP90 conformation were corroborated by cross-linked pairs located at the interfaces between NTD/MT (HSP90α: Lys293-Lys363) and MD/CTD (HSP90α: Lys444-Lys616; HSP90β: Lys435-Lys607) (Fig. 2b). These interfaces undergo significant reorientation during the HSP90 conformational cycle, implying a distinct HSP90 conformation favored by PU-H71 compared to GA. Lys444 in HSP90α (Lys435 in HSP90β) and Lys616 in HSP90α (Lys607 in HSP90β) are positioned either within the middle of the MD or in proximity to the central axis of the HSP90 homodimer (Fig. 2c). The distance between these lysine residues can provide insights into the relative placement of the monomer arms in specific HSP90 conformations (e.g., 20 Å in closed-like conformations; 29 Å in open-like conformations). The lower cross-linking percentage observed for Lys444 and Lys616 in HSP90α (Lys435 and Lys607 in HSP90β) in GA-favored HSP90 suggests a longer distance (29 Å) between them, supporting GA’s preference for binding to an open-like conformation. In contrast, the moderate cross-linking percentage detected for these residues in PU-H71-favored HSP90 implies a medium distance (20 Å) between them, favoring a closed-like conformation enriched in epichaperomes (Fig. 2c).

Additionally, a third pair of cross-linked residues (Lys293 and Lys363 in HSP90α) supports this notion. Located near the interface between the NTD and the MD, their positions are sensitive to the ligand binding state of the NTD, leading to changes in the relative positioning of secondary structures near the NTD/MD interface and altering the distance between Lys293α and Lys363α. Consistent with the cross-linked pair at MD/CTD interface, a closed-like conformation (16 Å) in PU-H71 bound HSP90 will be more amenable than an open-like conformation (13 Å) in GA-bound since the short distance might have limited the location of side-chains for cross-linking reactions.

In summary, our CX-MS data, supported by several cross-linked residue pairs situated in structurally distinct regions, the nucleotide-binding pocket, and the NTD/MD and MD/CTD interfaces, shed light on the conformation adopted by HSP90 within epichaperomes. These findings underscore the notion that an enrichment of the closed-like conformation of HSP90 in specific cellular environments favors the formation of epichaperomes.

### Specific PTMs support HSP90 incorporation into epichaperomes

**Figure 3.**
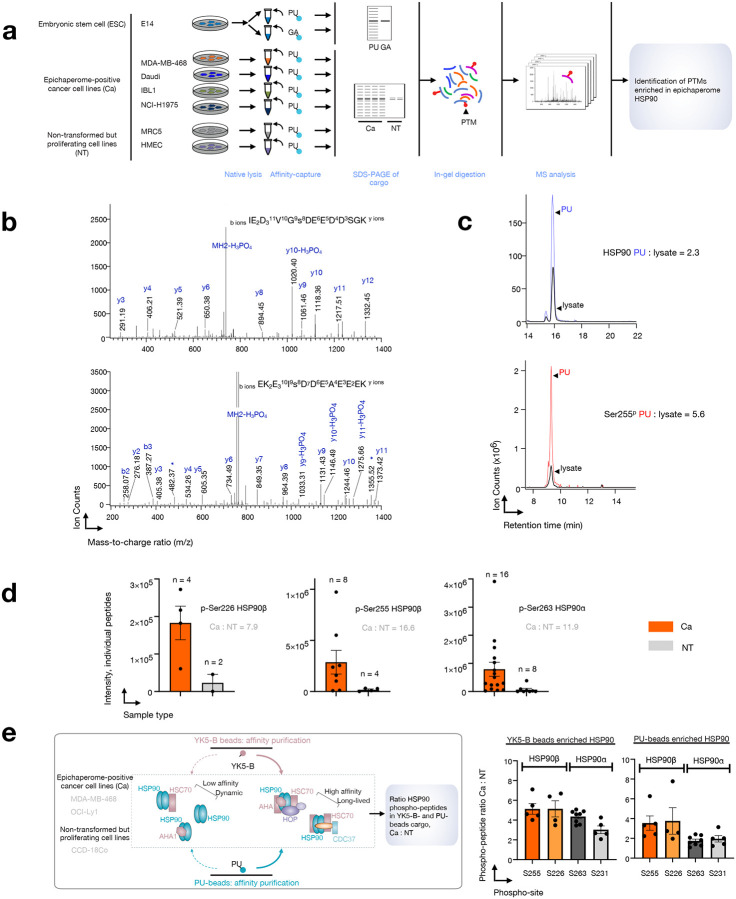
Phosphorylation of key residues located in the charged linker supports HSP90 incorporation into epichaperomes. **a** Experiment outline and expected outcomes. **b** Tandem MS spectra of HSP90 Ser226 (bottom) and Ser255 (top) phosphorylated peptides are presented, supporting the sequence and phosphorylation site identification. **c** Comparison of the extracted ion chromatogram of HSP90 Ser255 phosphopeptide in the PU-bead cargo (red trace, left panel) and ESC lysate (black trace, left panel) with a representative unmodified tryptic peptide in the PU-bead cargo (blue trace, right panel) and ESC lysate (black trace, right panel). **d** Ion intensity values of all phosphopeptides and the ratio of mean peptide intensity for each phosphosite in the samples described in panel a (n = 4 Ca and n = 2, NT). **e** Ratio of individual peptide intensity for each phosphosite in the samples described in the schematic (S255 n = 5; S226 n = 4; S263 n = 8; S231 n = 5). Source data are provided as Source Data file and as Supplementary Data 3,6.

To uncover the factors that facilitate the enrichment of the epichaperome-favoring HSP90 conformation, we conducted a comprehensive examination of the HSP90 pools isolated by PU-H71 and GA, searching for potential differences. Notably, we identified several peptides phosphorylated on Ser231 and Ser263 in HSP90α (Ser226 and Ser255 in HSP90β) exclusively in the PU-H71 cargo from ESCs (Fig. 3a,b and **Supplementary Data 3**). High-quality MS/MS spectra (illustrated for Ser226 and Ser255 phosphopeptides in HSP90β, Fig. 3b) coupled with precise mass accuracy allowed for the unequivocal identification of the peptide sequences and the phosphorylation sites. In contrast, these phosphorylated peptides were notably absent in substantial quantities in the GA cargo (**Supplementary Data 3**).

Subsequently, we performed label-free quantitation of these phosphopeptides using ion intensity measurements and observed a significant enrichment in the PU-beads cargo, particularly in the case of Ser255 of HSP90β. For instance, the Ser255 phosphopeptide displayed a nearly three-fold enrichment in the PU-H71 cargo compared to the lysate, after protein loading normalization using a representative tryptic peptide (Fig. 3c).

To gain further insights, we leveraged previously reported MS datasets of PU-H71-isolated cargo from epichaperome-positive cancer cells^13,19^, including MDA-MB-468 (triple negative breast cancer), Daudi (Burkitt’s lymphoma), IBL-1 (AIDS-related immunoblastic lymphoma), and NCI-H1975 (non-small cell lung carcinoma), as well as from non-transformed (NT) proliferating cells in culture (e.g., MRC5, lung fibroblast and HMEC, mammary epithelial cells). This analysis revealed that phosphorylation of these serine residues is also enriched in cancer cells when compared to NT cells (Ca:NT S255 = 16; S226 = 8; S263 = 12, Fig. 3d) establishing it as a hallmark of both ESC and cancer epichaperomes. This observation further supports the idea of a shared structural and architectural foundation for epichaperomes among ESCs and cancer cells.

As HSP90 is found alongside HSC70 in epichaperomes, we conducted an additional confirmatory experiment. Here, we used YK5-B, a biotinylated probe that binds to HSC70 in epichaperomes, and thus captures HSP90 in epichaperomes via HSC70^19^. PU-H71 and YK5-B were used to isolate cargo from epichaperome-positive cancer cells, including MDA-MB-468 and OCI-Ly1 (breast cancer and diffuse large B-cell lymphoma, respectively), as well as from CCD-18Co colon cells in culture (i.e., non-transformed proliferating cells in culture). We found that the Ser255 and S226 phosphopeptides of HSP90β were nearly four to five times more abundant in epichaperome-positive cancer cells compared to non-transformed proliferating cells in culture, for both the PU-cargo and the YK5-B cargo. Similar enrichment was noted for Ser263 and Ser231 in HSP90α (Fig. 3e). This analysis, thus, using both PU-H71 and YK5-B probes across diverse cell types, underscores the robustness of our observations and reinforces the role of phosphorylation in the acidic linker in shaping HSP90 within epichaperomes.

In light of these findings, made with two distinct probes and observed in ESCs, five cancer cell lines, each representative of a distinct cancer type, and of three non-transformed, but proliferating, cells in culture, it is evident that the epichaperome-specific agents target a subpopulation of HSP90 characterized by high phosphorylation levels in the acidic linker between the NTD and the MD, and this subpopulation predominantly assumes a closed-like conformation. In conjunction with PU’s preference for HSP90 within epichaperomes, and substantiated by YK5-B, a probe that binds epichaperomes via HSC70, these results strongly indicate that phosphorylation at these two serine residues is a key driver for HSP90 incorporation into epichaperomes and, consequently, for epichaperome formation.

### Specific PTMs drive epichaperome formation and function

To explore whether the phosphorylation of these serine residues plays a pivotal role in driving, rather than merely resulting from, epichaperome formation, we next studied the phosphomimetic (HSP90β^S226E,S255E^) and the non-phosphorylatable (HSP90^S226A,S255A^) mutants.

Notably, these serine residues are located within an intrinsically disordered region (IDR) of HSP90 (**Supplementary Fig. 5**). IDRs are pivotal elements in the intricate network of protein-protein interactions (PPIs). These regions lack a fixed three-dimensional structure, granting them exceptional flexibility. This structural adaptability enables proteins containing IDRs to assume various conformations in response to specific cellular contexts or binding partners. Such adaptability plays a crucial role in facilitating context-dependent involvement in distinct PPIs. In the case of HSP90, these serine residues within the IDR may alter the dynamics and structure of the charged linker, contributing to stabilizing the epichaperome-enabling conformation of this chaperone, and in turn facilitating epichaperome formation.

**Figure 4.**
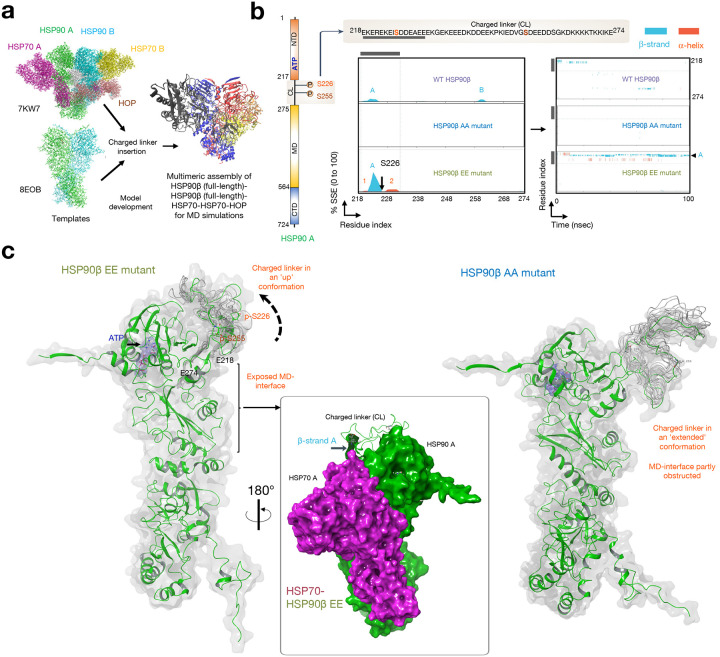
Phosphorylation of key residues located in the charged linker of HSP90 leads to a conformational shift in the linker, exposing the middle domain of the protein. **a** Model of the HSP90-HSP90-HSP70-HSP70-HOP assembly used for the molecular dynamics simulations. A and B, protomers A and B, respectively. **b** Protein secondary structure elements (SSE) like alpha-helices and beta-strands of the charged linker of protomer A of ATP-bound HSP90 monitored throughout the MD simulation. WT (HSP90 S226/S255), phosphomimetic (HSP90 S226E/S255E) and non-phosphorylatable (HSP90 S226A/S255A) mutants were analyzed. The plot on the left reports SSE distribution by residue index throughout the charged linker and the plot on the right monitors each residue and its SSE assignment over time. Schematic illustrating the primary structure of the full-length HSP90 with color-coded domains is also shown: NTD, N-terminal domain; MD, middle domain and CTD, C-terminal domain. The charged linker (CL) and the location of the two key serine residues are also shown (top inset). The gray bar indicates the CL segment encompassing residues 218 to 232. **c** Cartoon representation of ATP-bound HSP90 protomer A in assemblies containing the phosphomimetic (HSP90 S226E/S255E) or the non-phosphorylatable (HSP90 S226A/S255A) mutants is shown. Green, reference trajectory; gray, representative trajectories of n = 1,000. The inset illustrates the surfaces available for the interaction between HSP90 A and HSP70 A when the CL is in the ‘up’ conformation. A blue arrow indicates the location of the key beta-strand in the charged linker. See also Supplementary Figs. 5 and 6.

To explore this hypothesis, we conducted computational analyses to investigate the impact of each mutation on the flexibility of the charged linker (Fig. 4a–c). We constructed a model of the putative epichaperome core - namely the ~300 kDa assembly, see Fig. 1 - based on the cryo-EM structure of a multimeric HSP90 assembly (PDB: 7KW7). This structure represented 2xHSP90α, protomer A and B, bound to 2xHSP70 and 1xHOP. To create the model, we substituted HSP90 with human HSP90β using the closed-state cryo-EM structure (PDB: 8EOB). Additionally, we computationally inserted the charged linker, which was missing in the cryo-EM structures (Fig. 4a).

We conducted all-atom molecular dynamics simulation of this pentameric protein assembly, with each system containing all the components along with either the EE, AA, or WT HSP90 – in both protomers. These simulations are intended to qualitatively explore the immediate response of the assembly to the perturbation induced by mutations and not to provide an extensive characterization of the assemblies’ dynamics. By using a comparative MD-based approach we explore how short-term changes in the structural dynamics of different components within a large assembly may influence the emergence of states relevant for assembly stabilization. The underlying premise is that nanosecond timescale residue fluctuations in regions specifically responsive to certain states may facilitate large-scale rearrangements that underlie functional changes.

These simulations revealed that the structure and conformation of the charged linker were sensitive to the phosphorylation of the serine residues. In the pentameric assembly containing the phosphomimetic EE mutant (i.e., HSP90^S226E/S255E^), the linker of HSP90, protomer A, had a high probability of forming a β-strand bordering the Ser226Glu residue (2.1% of β-strand A). This strand remained stable over the duration of the simulation. This β-strand’s formation significantly decreased in the pentameric assembly containing the wild-type (WT, i.e., HSP90^S226/S255^) protein (0.4% of β-strand A), with no secondary structure element found in the assembly containing the AA (i.e., HSP90^S226A/S255A^) mutant (Fig. 4b). Notably, ATP binding, but not ADP binding, favored a charged linker with a high content of β-strand A formation (2.1% vs. 0.3%, respectively, in the EE mutant) (Fig. 4b and **Supplementary Fig. 6a)**. This finding emphasizes that the observed changes in the EE mutant were not merely due to the addition of charged residues; they were intricately tied to the phosphorylation status and the specific context, including the nucleotide environment permissive of the specific HSP90 conformation (i.e., closed-like). Intriguingly, the strategic formation of β-strand A not only stabilized the charged linker but also induced a conformational switch, flipping it into an ‘up’ conformation, thereby fully exposing the middle domain of HSP90, where HSP70 binds (Fig. 4c, see HSP90 protomer A – HSP70 interface). While other stabilized structural elements were observed in the analyzed assemblies containing either the WT or the mutant HSP90s, no other had a similar conformational effect on the charged linker as we observed for the β-strand A (see the effect of α-helices 1 through 6 in **Supplementary Fig. 6a,b**).

**Figure 5.**
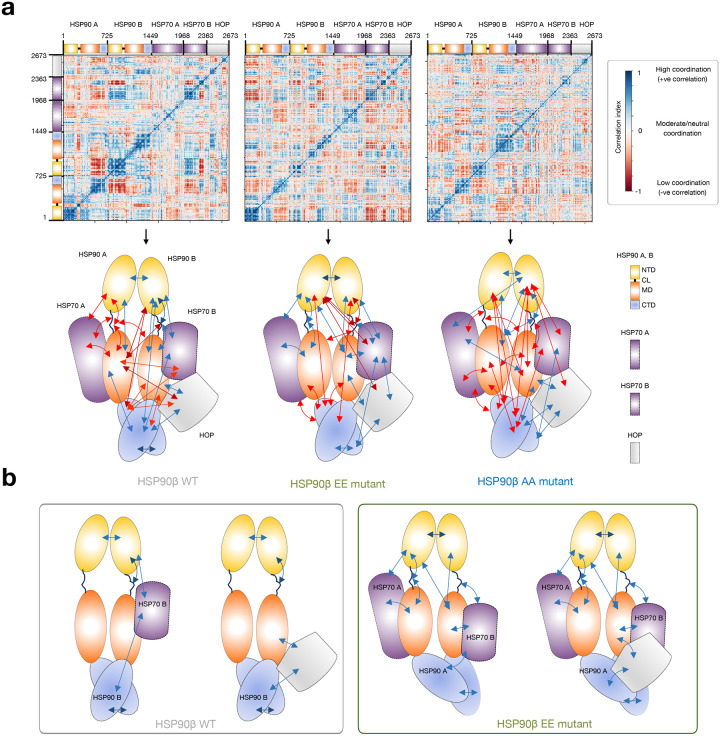
Phosphorylation of key residues located in the charged linker of HSP90 facilitates assembly motions conducive to epichaperome core formation. **a** Calculated dynamic cross-correlation matrix of Cα atoms around their mean positions for 100 ns molecular dynamics simulations. ATP-bound WT (HSP90 S226/S255), phosphomimetic (HSP90 S226E/S255E) and non-phosphorylatable (HSP90 S226A/S255A) mutant-containing HSP90-HSP90-HSP70-HSP70-HOP assemblies were analyzed. The cartoon below captures the key motions among the different domains of the individual assembly components. Extents of correlated motions and anti-correlated motions are color-coded from blue to red, which represent positive and negative correlations, respectively. The assembly contains two full-length HSP90beta proteins (protomer A and protomer B). The two HSP70 proteins (HSP70 A and HSP70 B) and the HOP protein are of sizes reported, and as per the constructs used in 7KW7. **b** Cartoon showing assemblies that are preferentially formed when the HSP90 charged linker is either phosphorylated (as in the EE mutant) or not phosphorylated (as in the WT protein).

We conducted dynamical residue cross-correlation analyses to explore how different protein units or subdomains in the pentameric 2xHSP90–2xHSP70-HOP assemblies, featuring either the WT (HSP90^S226/S255^) or mutant (HSP90^S226E/S255E^ or HSP90^S226A/S255A^) HSP90s, correlate in their motions throughout the simulation (Fig. 5a,b). This analysis aimed to reveal how individual components move in relation to each other. Positive dynamical cross-correlations spanning different components of the assembly within the large epichaperome core may indicate enhanced cooperative motions, suggesting increased interactions that contribute to the stability of the assembled structure. Previous studies have employed similar analyses to investigate how ligand-induced modulations influence the overall flexibility of HSP90 assemblies, facilitating progress along the chaperone cycle, thereby supporting feasibility of this approach^50^.

Indeed, we observed the highest correlation among the components in assemblies containing the HSP90 EE phosphomimetic, mimicking the case where the charged linker is phosphorylated, followed by the WT, and then the non-phosphorylatable HSP90 AA mutant (Fig. 5a). Notably, the coordinated movements observed in the assemblies containing the HSP90 phosphomimetic strongly support the idea that the HSP70-HSP90-HSP90-HSP70 or HSP70-HSP90-HSP90-HSP70-HOP assemblies can be preferentially stabilized when the HSP90 charged linker is phosphorylated (Fig. 5b). This observation aligns with the prominent ~300kDa band observed for the epichaperome core in native PAGE (see Fig. 1 showing HSP90 assemblies favored by PU-H71).

In contrast, in the WT HSP90 assembly, coordinated movements were primarily observed between the two HSP90 protomers, within HSP90, and between HSP90 and HSP70 and HOP, specifically through HSP90 protomer B (Fig. 5a,b). These movements are more consistent and favorable in the context of HSP90-HSP90-HSP70 or HSP90-HSP90-HOP assemblies. This observation implies that the major, broad ~242 kDa band detected by the HSP90 antibody – representing the primary HSP90-containing assembly observed in differentiated ESCs (Fig. 1) and in non-transformed cells^13–15,17,20^ – may consist of such assemblies, along with HSP90 homo-oligomers.

In summary, both MS evidence and computational models converge to support the conclusion that phosphorylation of the charged linker is a crucial contributor to epichaperome assembly, emphasizing its role in shaping not only HSP90, but also the stability and dynamics of the epichaperome structure.

**Figure 6.**
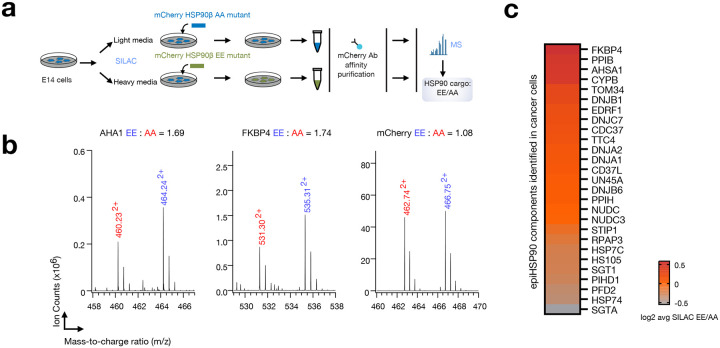
Immunopurification reveals increased presence of epichaperome-specific co-chaperones in phosphomimetic HSP90 complexes compared to non-phosphorylatable complexes. **a** Experiment outline and outcomes. **b** Representative spectra (n = 3 independent experiment) of proteins co-purified with the phosphomimetic HSP90^S226E,S255E^ (EE, blue) and non-phosphorylatable HSP90^S226A,S255A^ (AA, red) mutants. **c** Heatmap showing the identity of chaperone and co-chaperones identified as epichaperome components in cancer cells (as per Rodina et al. Nature 2016) and enriched in the affinity purified HSP90^S226E,S255E^ mutant. Scale bar, log2 average SILAC values EE/AA (n = 3). Source data are provided as a Source Data file and as Supplementary Data 4.

Next, we carried out an extensive biochemical and functional analysis to reinforce these findings. Given the well-established tight association between HSP90 and other chaperones and co-chaperones in epichaperomes^13,19,20,51^, our focus shifted to a comprehensive evaluation of chaperone and co-chaperone proteins co-purified with the phosphomimetic (HSP90β^S226E,S255E^) and non-phosphorylatable (HSP90^S226A,S255A^) mutants. Our strategy involved the purification of protein complexes containing N-terminally mCherry-tagged HSP90β in ESCs while retaining the endogenous WT HSP90 proteins. Distinctly labeled ESCs (i.e., labeled with heavy or light isotope lysine and arginine) expressing either the phosphomimetic or non-phosphorylatable mutant were subjected to immunoprecipitation (IP), followed by SDS-PAGE separation and quantitative analysis via MS to determine protein abundance (Fig. 6a–c; **Supplementary Data 4**). It is worth noting that we performed IP separately for the phosphomimetic and non-phosphorylatable mutants to minimize subunit exchange during IP^52^, thereby enhancing our ability to detect changes in co-chaperone binding more accurately than previous studies^53^.

We found co-chaperones were among the most abundant copurifying proteins, and most co-chaperones reported to participate in epichaperome formation^13,19^ displayed prominent changes in the phosphomimetic mutant (Fig. 6b,c). The increased presence of epichaperome-specific co-chaperones (such as AHA1 and FKBP4)^13^ in phosphomimetic complexes compared to non-phosphorylatable complexes highlights a stronger association with Ser226^P^/Ser255^P^ HSP90 as opposed to the non-phosphorylatable protein. However, we observed a slight reduction in the levels of HSC70 and HOP within phosphomimetic complexes. This decrease is potentially associated with specific subpopulations of HSP90 complexes that become more prevalent when the non-phosphorylatable Ala mutant is overexpressed in cells. The introduction of two Ala residues in the unstructured linker region of HSP90 may prompt the recruitment of HSC70 and HOP, chaperones recognized for their ability to bind unstructured unfolded protein stretches^54^. It is important to note that these assemblies are distinct from epichaperomes. Due to the anti-mCherry antibody capturing the entirety of the tagged HSP90, differentiation between specifically epichaperome-related HSP90 and a mixture of epichaperomes and other pools becomes challenging.

**Figure 7.**
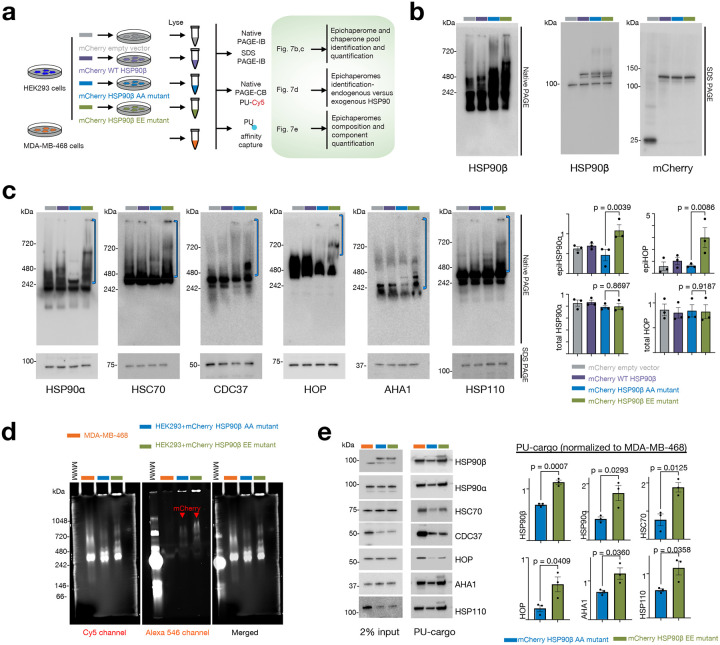
Phosphorylation of key residues located in the charged linker supports HSP90 incorporation into epichaperomes. **a** Overview of the experimental design and expected outcomes. **b** Analysis of transfection efficacy in cells transfected with HSP90β mutants, as indicated in panel a. **c** Detection of epichaperome components (chaperones and co-chaperones) through SDS-PAGE (bottom, total protein levels) and native-PAGE (top), followed by immunoblotting. Blue brackets indicate the approximate position of epichaperome-incorporated chaperones. Data are presented as mean ± s.e.m., n = 3, one-way ANOVA with Sidak’s post-hoc, EE vs AA. **d** Visualization of HSP90 in epichaperomes using the PU-TCO probe clicked to Cy5 (left) and the mCherry tag (middle). Right, merged images. MWM, molecular weight marker. **e** Detection and quantification of epichaperome components through PU-beads capture as indicated in panel a. Protein amount loaded for ‘Input’ represents 2% of the protein amount incubated with the beads. Data are presented as mean ± s.e.m., n = 3, unpaired two-tailed t-test. Gel images are representative of three independent experiments. Source data are provided as Source data file.

To address these limitations, we adopted a multi-pronged approach. Firstly, we utilized immunoblotting with native cognate antibodies for chaperone assemblies retained on native PAGE, coupled with chemical blotting using PU-probes. Additionally, we employed affinity capture with PU-probes to quantify the amount of epichaperome components under each condition (Fig. 7a). For these experiments, we transfected cells with the phosphomimetic (HSP90β^S226E,S255E^, EE mutant) and with the non-phosphorylatable (HSP90^S226A,S255A^, AA mutant) mutants, as well as with HSP90β WT or mCherry-tag only for control purposes. In this study, we chose human embryonic HEK293 cells as our cell model since they exhibit intermediate epichaperome expression levels (i.e., medium expressor, **Supplementary Fig. 7**), making them suitable for studying epichaperome dependence. We confirmed comparable transfection efficiency for each construct, with the tagged HSP90β protein expressed in addition to the endogenous HSP90β (Fig. 7b).

Our findings revealed that cells expressing the EE mutant exhibited higher levels of epichaperomes compared to those expressing the AA mutant, as evidenced by immunoblotting of various epichaperome components (including HSP90α, HSC70, CDC37, AHA1, HOP, and HSP110) (Fig. 7c, native PAGE) and chemical blotting with the PU-Cy5 epichaperome probe (Fig. 7d). Notably, there was no significant change in the overall concentration of these proteins in association with their incorporation into epichaperomes (Fig. 7c, SDS PAGE). Epichaperome isolation using PU-beads as an affinity purification probe also revealed significantly greater incorporation of chaperones, including mCherry-HSP90β, and co-chaperones into epichaperomes in cells expressing the EE mutant compared to those containing the AA mutant HSP90 (Fig. 7e), with no substantial alterations observed in cells containing the control vectors (**Supplementary Fig. 8a**). In contrast, overexpression of wild-type HSP90 in HEK293 cells had a minimal impact on endogenous epichaperomes (Fig. 7c, native PAGE, and **Supplementary Fig. 8a**, PU-beads capture). This observation aligns with previous reports^13^ suggesting that factors beyond chaperone concentration play a pivotal role in driving HSP90 incorporation into epichaperomes. Notably, cargo isolated on the control probe (control beads, **Supplementary Fig. 8b**) showed no detection of HSP90.

**Figure 8.**
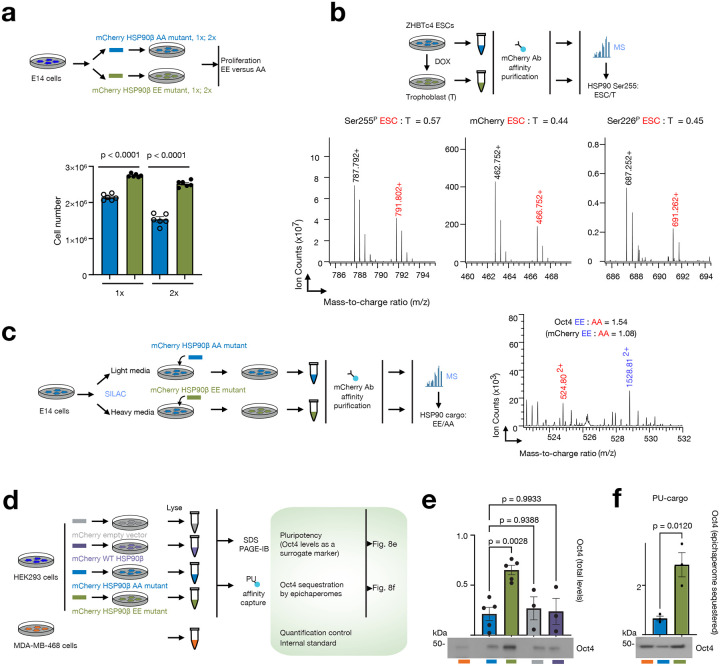
Phosphorylation of key residues located in the HSP90 charged linker favors ESC proliferation and self-renewal potential. **a** ESC proliferation at 60 h post-transfection in E14 cells transfected with either the phosphomimetic HSP90βS226E,S255E (EE) or the nonphosphorylatable HSP90S226A,S255A (AA) mutant. Medium (1x) or high (2x) plasmid concentrations were employed. Data are presented as mean ± s.e.m., n = 6, one-way ANOVA with Sidak’s post-hoc, EE vs AA. **b** Representative spectra (n = 3 independent experiments) of phosphopeptides, S255P (left) and S226P (right), and a representative unmodified tryptic peptide (middle) in mCherry-tagged WT HSP90β affinity-purified from ESC (red) or differentiated trophoblast (black) cells. **c** Representative spectra (n = 3 independent experiments) of a tryptic peptide from Oct4 protein co-purified from ESCs labeled with heavy or light isotope lysine and arginine expressing either the phosphomimetic (EE) or the non-phosphorylatable (AA) HSP90 mutant. Quantitative analysis via mass spectrometry (MS) to determine protein abundance is shown. **d** Overview of the experimental design and expected outcomes (panels e,f). **e,f** Detection and quantification of Oct4 protein expressed in cells transfected with the indicated HSP90 mutants or vector control (panel e) and sequestered into the epichaperome platforms (identified through PU-beads capture, panel f). (e) Data are presented as mean ± s.e.m., n = 5 AA, n = 5 EE, n = 3 WT, n = 3 empty vector, one-way ANOVA with Dunnett’s post-hoc, EE vs AA, WT vs AA, empty vector vs AA. (f) Data are presented as mean ± s.e.m., n = 3, unpaired two-tailed t-test. Source data are provided as Source Data file and Supplementary Data 5.

**Figure 9.**
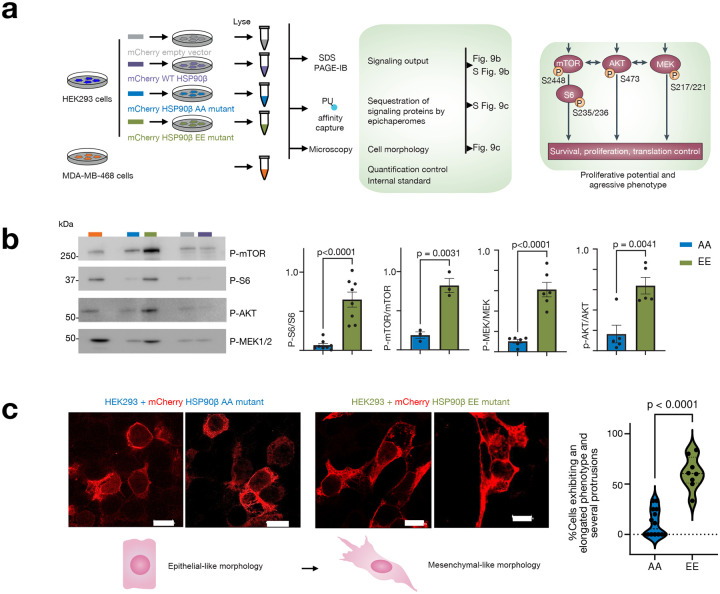
Regulation of epichaperome processes in ESC and cancer cells hinges on the specific phosphorylation events occurring at key residues within HSP90’s charged linker. **a** Overview of the experimental design and expected outcomes. **b** Detection and quantification of proteins involved in transducing signaling events that lead to cell proliferation, survival, and protein synthesis control. See Supplementary Fig. 9 for total protein levels and levels sequestered into epichaperomes. Data are presented as mean ± s.e.m., p-S6 n = 8; p-mTOR n = 3; p-MEK1/2 n = 6; p-AKT n = 5, unpaired two-tailed t-test. **c** Confocal microscopy shows morphological differences between the cells transfected with either the AA or the EE HSP90 mutant. Micrographs are representative of 96 cells for EE and 62 cells for AA. Scale bar, 10 μm. Data are presented as mean ± s.e.m., n = 8 wells for EE, n = 14 wells for AA, unpaired two-tailed t-test. Source data are provided as Source data file.

We further established the dependency of epichaperome function, beyond its formation, on the phosphorylation of HSP90 serine residues (Fig. 8,9). A key characteristic shared among high epichaperome-expressing cells in PSC, CSC, and cancer cells is the hyperactivity of the transcription factor c-MYC^13,25–27^. In cancer, c-MYC is frequently overexpressed or mutated, resulting in sustained activation, which drives uncontrolled cell proliferation^55^. In ESCs, c-MYC plays a crucial role in maintaining pluripotency and self-renewal, crucial for preserving the undifferentiated state of ESCs^56^. We therefore investigated the impact of HSP90β Ser226^P^/Ser255^P^ on cellular behaviors such as self-renewal and proliferation.

To assess proliferation, ESCs were transfected with plasmids containing either the phosphomimetic (HSP90β^S226E,S255E^) or non-phosphorylatable (HSP90β^S226A,S255A^) mutant. Notably, ESCs transfected with the HSP90β phosphomimetic mutant displayed a significantly higher proliferative rate (P<0.0001, >25%) compared to those transfected with the non-phosphorylatable variant, regardless of whether medium (1x) or high (2x) plasmid concentrations were employed (Fig. 8a). This observation lends support to the notion that HSP90β Ser226^P^/Ser255^P^, and consequently, epichaperomes, play a crucial role in ESC proliferation.

Differentiation of ESCs results in a decreased proliferative rate, as indicated by the doubling time of ZHBTc4 ES cells (~12 h) and trophoblast-differentiated cells (~25 h)^32^. Since differentiation is also closely associated with the disassembly of epichaperomes, we next examined the phosphorylation levels of HSP90β at Ser226 and Ser255 in cells with varying self-renewal capacities. We utilized the TET-repressible *oct4* mouse ESC line ZHBTc4, where the Oct4 expression is suppressed in the presence of doxycycline for ESC differentiation into trophoblast-like cells (Troph)^31^. In this experiment, we expressed WT mCherry-HSP90β in ZHBTc4 cells and quantified phosphopeptides in both ESCs and trophoblast cells following ESC differentiation (Fig. 8b, **Supplementary Data 5**). After normalizing the data to mCherry-HSP90β protein loading (middle panel, ES/Troph = 0.44), we observed a 30% higher phosphorylation of HSP90β at Ser255 in stem cells compared to differentiated cells (left panel, ES/Troph = 0.57). Phosphorylation levels of HSP90β at Ser226 appeared to remain unchanged under these experimental conditions after normalizing to protein loading (right panel, ES/Troph = 0.45).

Pluripotency hinges on crucial transcription factors like Oct4. Oct4 is widely recognized as one of the principal transcription factors governing the self-renewal of both pluripotent stem cells and cancer cells^57^. We find Oct4 interacts with epichaperomes in ESCs (**Supplementary Data 1**) and exhibits significant enrichment in the cargo captured with the Ser226/Ser255 phosphomimetic compared to the non-phosphorylatable HSP90 (**Supplementary Data 4**, Fig. 8c, 1.4-fold EE : AA). To validate the reliance of Oct4 on epichaperomes, we examined Oct4 levels in both MDA-MB-468 cancer cells and HEK293 cells transfected with the various HSP90 plasmids. Additionally, we utilized affinity capture with PU-probes (Fig. 8d–f and **Supplementary Fig.8a**). Notably, we observed that cells expressing the phosphomimetic EE mutant showed significantly elevated levels of Oct4, both overall (Fig. 8e) and within epichaperomes (i.e., those sequestered within the epichaperomes, Fig. 8f), compared to cells expressing the HSP90 AA mutant. No detectable differences were observed under control conditions (WT HSP90 and empty vector only) (**Supplementary Fig. 8a**). Additionally, Oct4 was sequestered by epichaperomes in MDA-MB-468 cells, supporting the idea that epichaperomes play a role in regulating pluripotency through both direct and indirect regulation of Oct4.

Epichaperomes play a pivotal role in supporting enhanced proliferation by altering the regulation of various proteins involved in cell signaling^3,13,19^. Higher epichaperome levels translate to a greater number of proteins being affected, resulting in increased signaling output^13,17,58^. We therefore next assessed the signaling output of cells transfected with the various HSP90 mutants. We observed a significantly heightened epichaperome-dependent impact on key signaling effector proteins involved in cell growth and proliferation (i.e., MEK, AKT, and mTOR) in cells expressing the HSP90 EE mutant compared to those expressing the AA mutant. This was evident in both the increased phosphorylation status of these effector proteins (Fig. 9a,b) and their enhanced recruitment to epichaperome platforms (**Supplementary Fig. 9a-c**) in cells expressing the EE mutant, as compared to those expressing the AA mutant. Importantly, these effects occurred without notable changes in the expression levels of the proteins (**Supplementary Fig. 9a, b**). No measurable differences were observed under control conditions (WT HSP90 and empty vector only) (Fig. 9b and **Supplementary Fig. 9a, b**).

Epichaperome formation fuels aggressive behaviors in cells^51,59^. Indeed, when observed under a microscope, we noted that, in comparison to cells expressing the non-phosphorylatable AA mutant (HSP90β^S226A,S255A^), those expressing the phosphomimetic EE mutant (HSP90β^S226E,S255E^) displayed a higher prevalence of cells with an elongated phenotype and several protrusions (Fig. 9a,b), supportive of a mesenchymal-like phenotype^60^. These morphological changes suggest a shift towards a more stem cell-like state, or a more aggressive phenotype in the context of cancer, in cells harboring the EE HSP90 mutant (i.e., with a high epichaperome load), a feature not observed in cells carrying the AA HSP90 mutant (i.e., not permissive of epichaperome formation).

**Figure 10.**
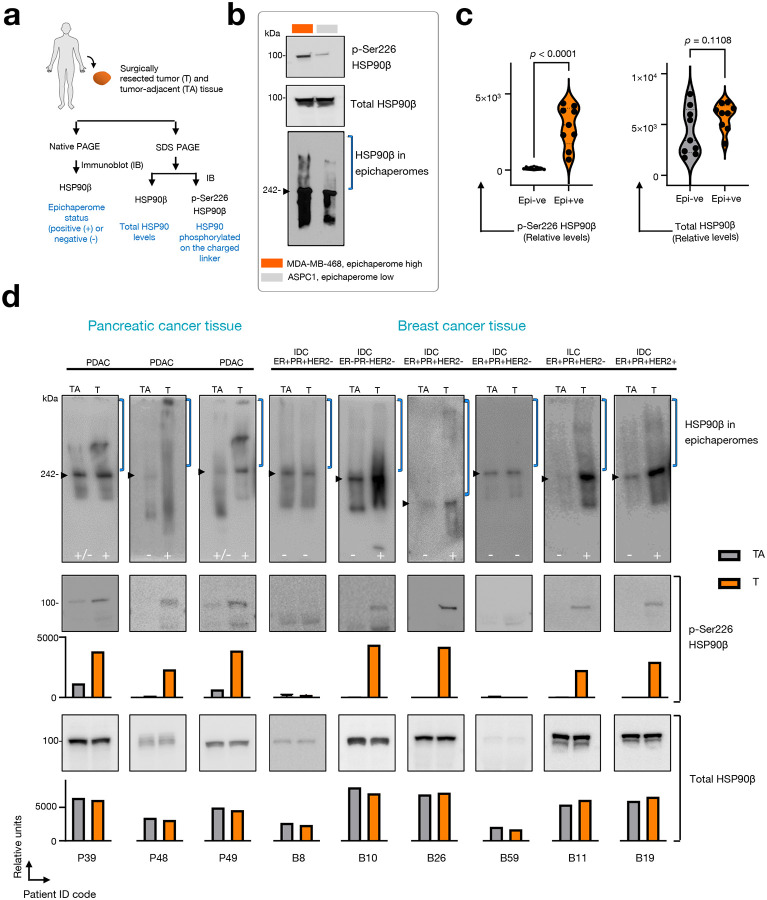
Human tissues positive for epichaperomes exhibit p-Ser226 HSP90β positivity, and conversely, those negative for epichaperomes show no or negligible p-Ser226 signal within HSP90’s charged linker. **a** Cartoon illustrating the processing of human tissue for biochemical analyses. Both tumor (T) and tumor adjacent (TA) tissues, determined by gross pathological evaluation to be potentially non-cancerous, were harvested and analyzed. **b** MDA-MB-468 breast cancer cells (epichaperome-high) and ASPC1 pancreatic cancer cells (epichaperome-low) served as controls for assessing p-Ser226 HSP90 levels. **c** The graph presents the relationship between epichaperome positivity and HSP90 Ser226 phosphorylation for tissues described in panel a. Data represent mean ± s.e.m., with n = 9 tumor (T) and n = 9 paired tumor-adjacent (TA) tissues classified based on epichaperome positivity or negativity, as determined by Native PAGE (see panel d); unpaired two-tailed t-test. **d** Detection of epichaperomes through native-PAGE (top), and of p-Ser226 HSP90 (middle) and total HSP90 (bottom) by SDS-PAGE, followed by immunoblotting, in tissues from the indicated patient specimens, as in panel a. Blue brackets indicate the approximate position of epichaperome-incorporated HSP90. Note: Obtaining genuinely “normal” tissue adjacent to tumors presents challenges, especially in the case of pancreatic tissue. The relatively small size of the organ and the nature of surgical procedures for pancreatic cancer often lead to the collection of normal samples in close proximity to the tumor. It’s crucial to acknowledge that, due to these challenges, we designate potentially normal tissue as tumor-adjacent tissue, recognizing that it may not entirely reflect a truly normal tissue state. PDAC, Pancreatic Ductal Adenocarcinoma; IDC, Invasive Ductal Carcinoma; ILC, Invasive Lobular Carcinoma; ER, Estrogen Receptor; PR, Progesterone Receptor. Source data are provided as Source data file.

Previous studies have found that irrespective of the tumor type, 60–70% of tumors contain HSP90-HSC70 epichaperomes^13,19^. Additionally, epichaperomes are known to specifically form in diseased tissue^3^. To assess whether our observations regarding the impact of the HSP90 charged linker, derived from cell models, extend to human patients and are not artifacts specific to cultured cells, we obtained surgical specimens from breast and pancreatic cancer surgeries (n = 18 tissues from 9 patients, Fig. 10a–d). Both tumor (n = 9) and tumor adjacent (n = 9) tissues, determined by gross pathological evaluation to be potentially non-cancerous, were analyzed for epichaperome levels using Native PAGE. Additionally, total HSP90β and phosphorylated HSP90β at Ser226 were assessed by SDS PAGE and immunoblotting with specific antibodies. To mitigate potential biases arising from varying HSP90 levels, each pair was normalized based on HSP90 concentration. Despite challenges in obtaining high-quality epichaperome profiles from surgical samples, a robust correlation emerged between epichaperome expression and Ser226 phosphorylation (Fig. 10c,d). Tissues positive for epichaperomes exhibited p-Ser226 HSP90β positivity, and conversely, those negative for epichaperomes showed no or negligible p-Ser226 signal.

Collectively, these multifaceted biochemical and functional lines of evidence establish a compelling connection between structural features in HSP90 and the processes of epichaperome formation and function. These findings lend robust support to the hypothesis that the regulation of epichaperome processes in ESC and cancer cells—encompassing critical factors such as proliferative potential, self-renewal capacity, plasticity, and signaling output—crucially relies on the specific phosphorylation events taking place at key residues within HSP90’s charged linker.

## DISCUSSION

The intricate network of protein-chaperone interactions within cells plays a critical role in maintaining protein homeostasis and cellular function. In recent years, the discovery of epichaperomes as specialized chaperone complexes in both cancer cells and pluripotent stem cells has opened new avenues for understanding chaperone biology. This investigation offers valuable insights into the structural and regulatory intricacies of epichaperomes, with particular attention to the pivotal role played by PTMs of HSP90 in orchestrating their formation and function.

A central discovery in this investigation is the recognition of specific PTMs on HSP90, especially at Ser226 and Ser255, as critical factors governing the assembly of epichaperomes. Our data reveal that phosphorylation of these serine residues enhances the association of HSP90 with other chaperones and co-chaperones, creating a microenvironment conducive to epichaperome formation. This finding underscores the significance of PTMs in regulating chaperone assemblies and highlights the potential of targeting these modifications for therapeutic intervention.

Chaperones appear to be highly susceptible to structural and functional regulation by a spectrum of PTMs. For example, PTMs of HSP90 provide an important regulatory element, modulating co-chaperone and client protein binding^61–65^, ATPase activity^66^, conformational cycle^62,65–67^, turnover^68^ and small molecule affinity^12,38^. Similar to minor changes in primary sequence, these PTMs likely regulate the access to and occupancy of key conformational states of HSP90 for in vivo processing of some essential clients. Our investigation pinpoints crucial PTMs that remodel the functional profile of HSP90, metamorphosing it from a protein-folding entity into epichaperomes, a platform orchestrating the reorganization of PPI networks for heightened cellular adaptability and proliferation.

Our study uncovered a fascinating aspect of PTMs in HSP90 within epichaperomes – phosphorylation events occur in an IDR of the protein. The strategic placement of these PTMs in the IDR holds profound significance, suggesting that they influence HSP90’s conformation and function beyond the traditional structured regions. This adaptability is crucial for HSP90’s participation in distinct PPIs, allowing it to stabilize the epichaperome-enabling conformation and restructure the interactions of numerous proteins in response to cellular stressors. Intriguingly, previous studies in yeast^69^, where the IDR was substituted with glycine-glycine-serine residues, align with our findings. These studies suggested that the charged linker (encompassing the IDR), influenced by the N-domain of HSP90, can adopt a structured form. This structured form, in turn, can stabilize interactions between specific HSP90 domains, influencing HSP90 dynamics, co-chaperone binding, and overall biological function, especially in conditions of cellular stress.

Changes in PPI networks play a fundamental role in cellular responses to stressors and the coordination of various biological processes^18^. These alterations, often induced by external stressors, are vital for the cell’s ability to adapt and function under different conditions. Notably, less than 10% of human PPIs remain unaffected by stress-induced perturbations, highlighting the widespread impact of cellular stress on the interactome. These changes, influenced by factors such as PTMs and protein conformation, are essential for species-specific adaptation and contribute to PPI network malfunctions observed in diseases.

One intriguing question is which kinase could phosphorylate HSP90 at these serine residues? A likely candidate is casein kinase II (CK2)^70,71^. CK2 is sequestered to epichaperomes in ESCs and in cancer cells^13^. Notably, CK2 is overexpressed in highly proliferative cells^72^ and plays a role in phosphorylating numerous protein substrates involved in cell proliferation and survival^73^. Moreover, the mutation of CK2 has been shown to abolish the viability of both PSCs^74^ and tumor cells^75,76^, indicating a potential direct link between epichaperome function and cellular physiology, possibly mediated by CK2 phosphorylation, which remains to be confirmed.

The implications of our study go beyond providing structural and mechanistic insights. We present compelling evidence that phosphorylation of HSP90 at Ser226 and Ser255 not only promotes epichaperome formation but also influences cellular behaviors, including proliferation and self-renewal. This suggests a direct link between epichaperome function and cellular physiology, particularly crucial in contexts such as cancer and stem cell maintenance, where robust proliferation and adaptation are vital.

Plasticity, a key characteristic associated with both ESCs and cancer cells^77^, is also implicated in our findings. The morphological changes observed in cells expressing the phosphomimetic HSP90 mutant—specifically, the higher prevalence of cells with an elongated phenotype and several protrusions—hint at a mesenchymal-like phenotype^60^. This phenotypic shift is often associated with increased plasticity and is indicative of a more stem cell-like state. Our findings suggest a potential role for epichaperomes in modulating this dynamic process of cellular transition between different phenotypic states.

The link between pluripotency and cancer is particularly intriguing. Cellular stress is increasingly recognized as a pivotal factor that can shift the balance between cellular pluripotency and the development of malignancies. The process of dedifferentiation, observed in regeneration in plants and some vertebrates, involves the deactivation of genes responsible for cell-specific functions, re-entry into the cell cycle, proliferation, and activation of pluripotency-associated genes^78^. Tumors also undergo dedifferentiation, where cancer cells revert to a less differentiated state, re-express stem cell genes like Oct4, leading to the emergence of cancer stem-like cells with enhanced metastatic potential and treatment evasion^79^. Our study proposes epichaperomes as significant mediators of changes in cellular identity, partly through Oct4.

The revelation of HSP90’s dysfunctional multimeric states carries implications for therapeutic interventions^3,16^. Instead of universally inhibiting all HSP90 pools, a paradigm shift comes to the fore with precision medicine strategies. The prospect of targeting specific pathologic conformations while preserving normal HSP90 functions emerges as a promising direction. This shift beckons researchers to navigate the intricate interplay of HSP90 conformations as they forge ahead in the quest for innovative therapeutic approaches. Our study also confirms the notion that small molecule HSP90 binders have distinct preference for HSP90 conformers in cells, reinforcing the finding that not all HSP90 inhibitors act equally well or equally selectively on specific disease-promoting HSP90 conformations or disease-associated HSP90 assemblies in comparison with HSP90 conformers found in normal cells. The first feature determines drug efficacy, whereas the latter influences the safety profile during administration.

In conclusion, our study unravels the intricate interplay between PTMs, conformational regulation, and biological functions of HSP90 within epichaperomes. These findings have implications for the development of novel therapeutic strategies targeting chaperone complexes in diseases characterized by epichaperome dysregulation, such as in cancers and neurodegenerative disorders. By deciphering the regulatory mechanisms underlying epichaperomes, we move one step closer to harnessing their potential for precision medicine and therapeutic intervention.

## METHODS

### Human biospecimens research ethical regulation statement

Surgical specimens were obtained in accordance with the guidelines and approval of the Institutional Review Board at Memorial Sloan Kettering Cancer Center, Biospecimen Research Protocol# 09–121, project title: Ex-Vivo Testing of Breast Cancer Tumors for Sensitivity to Inhibitors of Heat Shock Proteins and Signaling Pathway Inhibitors, S. Modi, PI, and Biospecimen Research Protocol# Protocol# 09–121, project title: Ex-Vivo Testing of Breast Cancer Tumors for Sensitivity to Inhibitors of Heat Shock Proteins and Signaling Pathway Inhibitors, S. Modi, PI, and Biospecimen Research Protocol# 14–091, project title: Establishment and Characterization of Unique Mouse Models Using Patient-Derived Xenografts. E. de Stanchina, PI. The source of samples consists of unused portions of surgical specimens that are taken for reasons other than research (i.e., for patients undergoing the procedures for medical reasons unrelated to need for research samples or to the nature of the research). No individuals were excluded on the basis of age, sex or ethnicity. Because breast cancer is a disease which overwhelmingly affects women, and is a disease that is generally not seen in children, the vast majority of breast cancer patients enrolled on protocol# 09–121 were females >18 years of age. Patient tissue samples were obtained with consent provided in written form. Samples were de-identified before receipt for use in the studies.

### Reagents and Chemical Synthesis

All commercial chemicals and solvents were purchased from Sigma Aldrich or Fisher Scientific and used without further purification. The identity and purity of each product was characterized by MS, HPLC, TLC, and NMR. Purity of target compounds has been determined to be >95% by LC/MS on a Waters Autopurification system with PDA, MicroMass ZQ and ELSD detector and a reversed phase column (Waters X-Bridge C18, 4.6 × 150 mm, 5 μm) eluted with water/acetonitrile gradients, containing 0.1% TFA. Stock solutions of all inhibitors were prepared in molecular biology grade DMSO (Sigma Aldrich) at 1,000× concentrations. The PU-TCO, PU-CW800 and YK5-B probes and relevant control probes, and the PU-beads and the control probes were generated using published protocols^13,19,35,80–85^ or as described in **Supplementary Notes 1**. The GA-biotin probe was purchased from Sigma (SML0985). Disuccinimidyl suberate (DSS) was acquired from ThermoFisher (21655).

### Cell lines and culture conditions

Cell line selection was not based on gender, sex or ethnicity. Cell lines were cultured according to the providers’ recommended culture conditions. Cells were authenticated using short tandem repeat profiling and tested for mycoplasma. The breast cancer cell line MDA-MB-468 (HTB-132, RRID: CVCL_0419), pancreatic cancer cell line ASPC1 (CRL-1682, RRID: CVCL_0152), non-small cell lung cancer cell line NCI-H1975 (CRL-5908, RRID: CVCL_1511), B lymphoblast cell line Daudi (CCL-213, RRID: CVCL_0008), lung fibroblast cells MRC5 (CCL-171, RRID:CVCL_0440), the colon cell line CCD-18Co (CRL-1459, RRID: CVCL_2379) and the Human Embryonic Kidney 293 (HEK293) cell line (CRL-1573, RRID: CVCL_0045) were purchased from ATCC. IBL-1 (RRID:CVCL_9638) was derived from an AIDS-related immunoblastic lymphoma^86^. Mammary epithelial primary cells HMEC (PCS-600–010) were purchased from Lonza. B-cell lymphoma cell line OCI-LY1 (RRID:CVCL_1879) was obtained from the Ontario Cancer Institute. E14 mouse ES cells^87^ were received as frozen ampules from TG Fazzio (U Mass Med School). Cells were feed-free and verified as of male mouse origin through sequencing. ZHBTc4 mouse ES cells^31^ were received from D. Levasseur (U of Iowa). Cells were cultured as ESCs without feeder cells in the absence of doxycycline. For hiPSC, healthy donor fibroblasts purchased from Coriell were reprogrammed using CytoTune Sendai viruses^34^.

### Mammalian cell culture and lysis

Mouse feeder-free embryonic stem cells (E14 or ZHBTc4 line) were grown on tissue culture plates coated with 0.2% gelatin. ESCs were cultured in Dulbecco’s Modified Eagle Medium (DMEM; Gibco 10829018) media supplemented with 10% fetal bovine serum (FBS, HyClone SH30070.03HI), 2 mM L-glutamine, 0.1 mM nonessential amino acids (Gibco 11140050), 100 U mL^−1^ penicillin/streptomycin (Gibco 15140122), 0.1 mM beta-mercaptoethanol (Sigma M6250), and 103 U mL^−1^ leukemia inhibitory factor (LIF). Cells are grown in 37°C/5% CO_2_ incubator with media change every 2 days, passaged or harvested when 60–80% confluent. After harvesting, cell pellets are washed with phosphate-buffered saline (PBS, GenClone 25–508) and flash frozen before storing in −80°C. For pull-down and chemical cross-linking experiments, frozen cells are thawed and lysed in Felts lysis buffer (20 mM HEPES pH 7.4, 50 mM KCl, 5 mM MgCl_2_, 0.01% NP-40) in the presence of protease inhibitors, phosphatase and deacetylase inhibitors.

### ESC and hiPSC differentiation

ZHBTc4 cells were differentiated into trophoblasts through Oct4 repression. Cells were seeded at a density of 2 × 10^5^ cells mL^−1^ and grown in media with added doxycycline at a final concentration of 200 ng mL^−1^ for 96 h before harvest. E14 cells were spontaneously differentiated using attached embryoid bodies (EB) culture. Briefly, cells were seeded at a density of 5 × 10^4^ cells/mL in sterile bacteriological petri dishes in differentiation media (ES media without LIF) and cultured in 37°C/5% CO_2_ incubator for 4 days to aggregate into EBs. When turned orange, media were changed. On day 4, EBs were transferred into tissue culture dishes (without gelatin) at a density of 100–200 EBs per 10 cm tissue culture dish. Attached EBs were cultured in differentiation media in 37°C/5% CO_2_ incubator for 14–18 days before harvest. hiPSC differentiated in midbrain dopaminergic neurons were a gift from Dr. Lorenz Studer. Cells were differentiated into midbrain dopamine neurons by a modified dual-SMAD inhibition protocol as described^20^. hESCs were dissociated into single cells using Accutase and plated at high density on Matrigel (BD). The cells were subjected to timed exposure to LDN193189 (100 nM, Stemgent), SB431542 (10 μM, Tocris), SHH C25II (100 ng mL^−1^, R&D), Purmorphamine (2 μM, Stemgent), FGF8 (100 ng mL^−1^, R&D) and CHIR99021 (CHIR; 3 μM, Stemgent) to induce midbrain floor plate precursors. For mDA neuron induction, floor plate precursors were maintained in mDA differentiation media containing Neurobasal/B27/L-Glut (NB/B27; Invitrogen) supplemented with CHIR (until day 13) and with BDNF (brain-derived neurotrophic factor, 20n mL^−1^; R&D), ascorbic acid (0.2 mM, Sigma), GDNF (glial cell line-derived neurotrophic factor, 20 ng mL^−1^; R&D), TGFβ3 (transforming growth factor type β3, 1 ng mL^−1^; R&D), dibutyryl cAMP (0.5 mM; Sigma), and DAPT (10 μM; Tocris). On day 20, cells were dissociated using Accutase and replated on dishes pre-coated with polyornithine (PO; 15 μg mL^−1^)/laminin (1 μg mL^−1^)/fibronectin (2 μg mL^−1^) in differentiation medium (NB/B27 + BDNF, ascorbic acid, GDNF, dbcAMP, TGFβ3 and DAPT). On day 30 of differentiation, cells were dissociated using Accutase and replated on dishes pre-coated with polyornithine (PO; 15 μg mL^−1^)/laminin (1 μg mL^−1^)/fibronectin (2 μg mL^−1^) in differentiation medium (NB/B27 + BDNF, ascorbic acid, GDNF, dbcAMP, TGFβ3 and DAPT) supplemented with 10 μM Y-27632 (until day 32). Two days after plating, cells were treated with 1 μg mL^−1^ mitomycin C (Tocris) for 1 h to kill any remaining proliferative contaminants. The mDA neurons were fed every 2 to 3 days and maintained without passaging until they were assayed at day 65. To prevent neurons from lifting off, laminin and fibronectin were supplemented into the media every 7–10 days.

### Cell culture and transfections

Monolayer cultures of MDA-MB-468 and HEK293 cells were grown in high glucose (4.5 g L^−1^) DMEM containing 10% FBS and 1× antibiotic and antimycotic (100×ABAM, GIBCO) in a 37°C incubator supplied with 5% oxygen-air atmosphere. For native electrophoresis, and in-gel fluorescence studies, 1 × 10^7^ cells were seeded in 100 mm dishes (Corning) at 70% confluency in DMEM supplemented with 10% FBS and 1×ABAM. Next day, spent medium was changed with fresh serum and antibiotic free DMEM for 1 h before performing transfections. Cells were transfected using lipofectamine 3000 (Invitrogen) with 4 μg of mCherry empty vector, mCherry-HSP90β-Wild type (mCherry-HSP90β-WT), mCherry-HSP90β-S226A, S255A mutant (mCherry-HSP90β-AA) or mCherry-HSP90β-S226E, S255E mutant (mCherry-HSP90β-EE) plasmids. See Supplementary Note 2 for plasmid sequences. Transfection mixtures were prepared in OptiMEM (Gibco). Post 6 h of transfection, medium was changed with 10% FBS and 1×ABAM supplemented DMEM. Cells were harvested in native lysis buffer for future analyses.

### Primary specimen processing

Frozen tumor and matched tumor adjacent tissues were cut into small pieces using surgical blades and weighed using a precision balance. 74 mg of tissue was homogenized in 200 μL of 1× native lysis buffer in 1.5 mL microtube homogenizer for each sample. Homogenization was performed on dry ice. Post homogenization samples were incubated on ice for 30 min followed by centrifugation at 12,000×g at 4°C for 15 min. Supernatant was collected, and protein quantification was done using BCA method. Samples were normalized using total HSP90β levels for each tissue pairs. An initial SDS-PAGE was run using 5 μg of total protein for each sample. Total protein loads were adjusted to ensure equal levels of total HSP90β in tumor and corresponding matched adjacent tissue. Samples were then processed for native PAGE and SDS-PAGE to check for HSP90β and p-Ser226 HSP90β as described below.

### Native gel electrophoresis and western blot

Native gel electrophoresis was performed as reported^88^. Namely, 1 × 10^7^ cells were lysed in 20 mM Tris pH 7.4, 20 mM KCl, 5 mM MgCl_2_, 0.01% NP40, and 10% glycerol buffer containing protease and phosphatase inhibitors (native lysis buffer), by a freeze-thaw procedure. Protein concentrations were measured by using the BCA assay according to the manufacturer’s protocol (Pierce^™^ BCA Protein Assay Kit, Thermofisher Scientific, Waltham, MA). One hundred micrograms (100 μg) of protein were loaded in 4 to 10% native gel and run using native 1× Tris-Glycine buffer (25 mM Tris, 192 mM glycine) at 4°C in a cold room at 125V. Following electrophoresis, proteins were transferred to PVDF membrane, by wet transfer (25 mM Tris, 192 mM glycine, 20% (v/v) methanol, 0.02% SDS) at 100V in the cold room. Membranes were then blocked for 1 h in 5% BSA in TBS/0.1% Tween 20. The blots were then probed with the following antibodies: HSP90β (SMC-107; RRID:AB_854214; 1:2,000) and HSP110 (SPC-195; RRID:AB_2119373; 1:1,000) from Stressmarq; HSC70 (SPA-815; RRID:AB_10617277; 1:1,000), and HOP (SRA-1500; RRID:AB_10618972; 1:1,000) from Enzo; HSP90α (ab2928; RRID:AB_303423; 1:6,000), AHA1 (ab56721, RRID:AB_2273725, 1:1000) from Abcam; CDC37 (4793; RRID:AB_10695539; 1:1,000), HOP (5670; RRID:AB_10828378; 1:1,000), from Cell Signaling Technologies. The blots were washed with TBS/0.1% Tween 20 and incubated with appropriate HRP-conjugated secondary antibodies: goat anti-mouse (1030–05, RRID: AB_2619742, 1;5,000), goat anti-rabbit (4010–05, RRID: AB_2632593, 1:5,000) and goat anti-rat (3030–05, RRID: AB_2716837, 1:5,000) (Southern Biotech, Birmingham, AL, USA).] The chemiluminescent signal was detected with Enhanced Chemiluminescence (ECL) reagent according to manufacturer’s instructions and visualized using Chemi Doc (Biorad) and analyzed using Image Studio Lite Version 5.2. (LI‐COR Biosciences). NativeMark unstained protein standard (Invitrogen, LC0725) was used to estimate molecular weight of protein complexes in native gel electrophoresis and Western blotting.

### SDS-PAGE and western blot

Proteins were extracted in 20 mM Tris pH 7.4, 20 mM KCl, 5 mM MgCl_2_, 0.01% NP40, and 10% glycerol buffer containing protease and phosphatase inhibitors (native lysis buffer), by a freeze-thaw procedure. Protein concentrations were measured by using the BCA assay according to the manufacturer’s protocol (Pierce^™^ BCA Protein Assay Kit, Thermofisher Scientific, Waltham, MA). Ten to thirty micrograms (10 to 30 μg) of total protein were subjected to SDS–PAGE, transferred onto PVDF membrane, by wet transfer (Towbin buffer: 25 mM Tris, 192 mM glycine, 20% (v/v) methanol) at 100V in cold room. Membranes were then blocked for 1 h in 5% BSA in TBS/0.1% Tween 20 and incubated overnight with the indicated antibodies. HSP90β (SMC-107; RRID:AB_854214; 1:2,000) and HSP110 (SPC-195; RRID:AB_2119373; 1:1,000) from Stressmarq; HSC70 (SPA-815; RRID:AB_10617277; 1:1,000), and HOP (SRA-1500; RRID:AB_10618972; 1:1,000) from Enzo; HSP90α (ab2928; RRID:AB_303423; 1:6,000), AHA1 (ab56721, RRID:AB_2273725, 1:1,000) from Abcam; p-MEK1/2 (S217/221) (9154; RRID:AB_2138017; 1:1,000), MEK1/2 (9122; RRID:AB_823567; 1:1,000), p-mTOR (S2448) (5536; RRID:AB_10691552; 1:500), mTOR (2983; RRID:AB_2105622; 1:1,000), CDC37 (4793; RRID:AB_10695539; 1:1,000), HOP (5670; RRID:AB_10828378; 1:1,000), p-S6 ribosomal protein (Ser235/236) (4858; RRID:AB_916156; 1:2,000), S6 ribosomal protein (2217; RRID:AB_331355; 1:3,000), Oct4 (2840, RRID:AB_2167691, 1:2,000), p-AKT (S473) (9271, RRID:AB_329825, 1:2000), AKT (4691, RRID:AB_915783, 1:3000), HSP70 (ADI-SPA-810, RRID:AB_10616513, 1:2000) from Cell Signaling Technologies, β-actin (A1978, RRID: AB_476692, 1:3000) from Sigma-Aldrich, and mCherry (PA5–34974, RRID:AB_2552323, 1:2,000) and p-Ser226 HSP90β (PA5–105480, RRID:AB_2816908, 1:1,000) from Fisher Scientific. The blots were washed with TBS/0.1% Tween 20 and incubated with appropriate HRP-conjugated secondary antibodies: goat anti-mouse (1030–05, RRID: AB_2619742, 1;5,000), goat anti-rabbit (4010–05, RRID: AB_2632593, 1:5,000) and goat anti-rat (3030–05, RRID: AB_2716837, 1:5,000) (Southern Biotech, Birmingham, AL, USA). The chemiluminescent signal was detected with ECL reagent according to manufacturer’s instructions and visualized using ChemiDoc MP imaging system (Biorad) and analyzed using Image Studio Lite Version 5.2. (LI-COR Biosciences). Thermo Scientific PageRuler Plus prestained protein ladder (Fisher Scientific, 26619) or Precision Plus protein standards (Bio-Rad, 161–0375) were used as size standards in protein electrophoresis and Western blotting.

### Coomassie and Ponceau S staining

Where indicated, gels after native PAGE or SDS-PAGE were washed with deionized water three times for 5 min and incubated with Coomassie G-250 stain (Bio-Rad) for 1 h. The gels were washed with water after to remove the excess of the dye and imaged. Where indicated, membranes after protein transfer were incubated with Ponceau S solution (Sigma) for 10 min, then were washed with water to remove the excess of the dye and imaged.

### Primary specimen analyses

Specimens were harvested as previously reported^89^. Briefly, the surgical team delivered specimens in tightly sealed, sterile, leak-proof bags without fixatives. This maintained specimens in their fresh state, crucial for downstream analyses. Fresh specimens underwent sterile harvesting by the pathologist or assistant, using laminar flow hoods. Harvesting times were meticulously recorded, kept under 30 minutes post-surgery to mitigate cold ischemia effects. Primary breast tumor specimens were selectively obtained from the index lesion’s periphery, avoiding central necrosis. Recognition criteria for necrotic tissue included color loss, softness, and demarcation from viable tissue. Normal breast tissue samples (e.g., normal dense/fibrous breast parenchyma) are taken from distant locations, at least 1 cm grossly away from the target lesion if feasible. In contrast, due to the relatively small size of the pancreas and the nature of surgical procedures, normal pancreas samples collected were typically in close proximity to the tumor. Whipple procedures typically involve the resection of the head of the pancreas, while distal procedures focus on the resection of the tail. Samples were initially stored in tubes with MEM and antibiotics and transported on wet ice to the laboratory immediately after procurement. Upon reaching the laboratory, samples were transferred to cryovials, ‘snap’ frozen, and stored at −80 °C for future molecular analyses.

### Chemical blotting

For in-gel blotting using PUTCO, cells were harvested in 20 mM Tris pH 7.4, 20 mM KCl, 5 mM MgCl_2_, 0.01% NP40, and 10% glycerol buffer containing protease and phosphatase inhibitors (native lysis buffer), by a freeze-thaw procedure. Protein concentrations were measured by using the BCA assay according to the manufacturer’s protocol (Pierce^™^ BCA Protein Assay Kit, Thermofisher Scientific, Waltham, MA). One hundred micrograms (100 μg) of protein were incubated with 1 μM of PUTCO in a total volume of 42 μL. Post 3 h of incubation samples were loaded in 4 to 10% native gel and run using native 1× Tris-Glycine buffer at 4°C in cold room at 125V. Following electrophoresis, the gel was incubated in 30 mL of 700 nM Cy5-Tetrazine containing ice cold 1× Tris-Glycine buffer at room temperature (RT) for 15 min for the click reaction to occur. After 15 min, the gel was washed thrice (5 min each) with ice cold 1× Tris-Glycine buffer. The gel was then imaged using ChemiDoc MP imaging system (Biorad). Alexa 546 channel (illumination: Epi-green, 520–545 nm excitation, Filter: 577–613 nm filter for green-excitable fluorophores and stains) was used to visualize mCherry-tagged species, and native page ladder (NativeMark^™^ Unstained Protein Standard, Cat. No. LC0725, Invitrogen^™^). The Cy5 channel (illumination: Epi-far red, 650–675 nm excitation, Filter: 700–730 nm filter for far red-excitable fluorophores and stains) was used for imaging PUTCO staining. Post capturing, the images from the two channels were merged to get the alignment of the bands with respect to the molecular weight ladder in Image Lab 6.1 (Bio-Rad). For in cell blotting using PU-CW800, E14 cells were plated at a seeding density of 1 × 10^6^ per 10 cm plate and grown for 44 h before treatment with either PU-CW800 or control fluorophore (SS27) at a concentration of 1 μM in culture media for 4 h while incubating at 37°C, 5% CO_2_. Following the treatment, cells were harvested and lysed by dounce homogenization in Felts lysis buffer (20 mM HEPES at pH 7.4, 50 mM KCl, 2 mM EDTA, and 0.01% NP40) supplemented with protease, phosphatase, and deacetylase inhibitors. Cell lysates were buffer exchanged with fresh Felts lysis buffer containing supplements to remove any unbound drug before loading into a native gel. For visualization of PU-CW800 fluorescence and total protein, 200 μg of cell lysate was loaded onto a 4–10% native gradient gel and resolved at 4°C for 5 h. Fluorescence was visualized on LI-COR Odyssey CLx using Image StudioTM Software (LI-COR Biosciences) and then total protein was visualized on the same gel using Coomassie Brilliant Blue R250 stain. Band(s) with observable fluorescent signal were then processed by in-gel digestion and analyzed for LC-MS/MS to identify major proteins.

### SILAC and ESC transfection

For metabolic labeling with SILAC (stable-isotope labeling of amino acid in cell culture), ESCs were cultured and passaged five times at 48 h intervals in media containing SILAC DMEM (Thermo Fisher 88364) supplemented with 13C- and 15N-labeled heavy L-arginine (84 mg L^−1^, Cambridge isotope CNLM-539-H) and L-lysine (146 mg L^−1^, Cambridge isotope CNLM-291-H) or supplemented with 12C- and 14N-labeled light L-arginine (Fisher BP2505100) and L-lysine (Fisher J6222522) amino acids for five passages to ensure complete stable isotope incorporation. For heterologous expression of HSP90 AA or EE mutants, cells were then reverse transfected with plasmid DNA using LipofectamineTM 3000 Transfection Kit (Invitrogen #L3000015) and incubated at 37°C, 5% CO_2_ for 72 h at which point they were harvested.

### Measurement of cell proliferation

E14 cells were transfected and incubated in 37°C/5% CO_2_ incubator for 24 h. Cells were then replated to 6-well plate at the same dilution factor for each transfection treatment condition and then returned to incubator. At 60 h post-transfection, cell proliferation was determined via cell count for all conditions.

### Confocal microscopy

HEK293 cells transfected with mCherry-HSP90β-AA or mCherry-HSP90β-EE plasmids were seeded at a density of 1.8 × 10^6^ cells mL^−1^ on coverslips in a monolayer in six well plates and then grown overnight for the cells to attach. Cover slips were mounted with ProLong^™^ Gold antifade mountant with DAPI. Imaging was done using Leica SP8 Stellaris microscope. Images were analyzed using Image J and Leica LAS X lite software. Cell morphology was manually inspected, and the percentage of cells exhibiting an elongated phenotype and several protrusions was calculated. Specifically, cells transfected with mCherry were assessed, and those displaying the described features were counted. The percentage was then determined based on the total number of mCherry-transfected cells observed.

### Chemical precipitation and cross-linking

The GA-affinity beads were prepared by incubating GA-biotin (Sigma SML0985) with Dynabeads M-280 Streptavidin (ThermoFisher 11205D) at 4°C for 2.5 h. The GA-bound beads were then incubated with cleared cell lysates or cross-linked cell lysates overnight at 4°C. For PU-beads affinity capture, cell lysates were incubated with PU-beads or control beads at 4°C for 3.5 h. Following incubation, bead conjugates were washed three times in lysis buffer before elution with sample buffer. The chemical cross-linking and HSP90 purification experiments were carried out in >3 replicates for both ligands. Samples were analyzed separately, and statistical significance was assessed.

### Chemical precipitation and immunoblotting

Cells were harvested in 20 mM Tris pH 7.4, 20 mM KCl, 5 mM MgCl_2_, 0.01% NP40, and 10% glycerol buffer containing protease and phosphatase inhibitors (native lysis buffer), by a freeze-thaw procedure. Protein concentrations were measured by using the BCA assay according to the manufacturer’s protocol (Pierce^™^ BCA Protein Assay Kit, Thermofisher Scientific, Waltham, MA). PU-beads and control beads were washed with the native gel buffer 3 times prior use. Post washing, 40 μL aliquots of the beads were distributed into the sample tubes. Five hundred micrograms (500 μg) of total protein in 300 μL final volume, adjusted with native lysis buffer were added. Samples were incubated for 3 h at 4°C on a rotor, followed by washing with native lysis buffer four times. Post washing, 30 μL of 5× Laemmli buffer was added to the beads and boiled at 95°C for 5 min. Ten micrograms (10 μg) of the lysates (2%) was used as input for the pull-down experiment. Samples were then centrifuged at 13,000 × g for 20 min and supernatant collected was loaded on to SDS-PAGE. The protein transfer and western blotting procedures were performed as described in SDS-PAGE and western blot section.

### IUPred analysis for disorder prediction

Sequence Preprocessing: The primary amino acid sequence of human HSP90β (P08238) and HSP90α (P07900) were extracted in FASTA format. These sequences served as the input for subsequent disorder prediction using the IUPred algorithm. Calculation of Disorder Scores: The IUPred algorithm utilizes energy potentials derived from pairwise amino acid interactions to assess the local structural propensities of each residue in the protein sequence. For each residue, IUPred computes a disorder score within the range of 0 to 1. A score of 0 suggests a higher likelihood of being ordered, while a score of 1 indicates a higher likelihood of being disordered. Threshold for Disorder Classification: To classify residues as either ordered or disordered, a threshold was applied to the calculated disorder scores. A common threshold of 0.5 was employed, designating residues with scores above 0.5 as disordered. The output of the IUPred analysis consisted of a disorder profile, providing disorder scores for each residue in the input protein sequence. Residues were categorized based on the applied threshold, facilitating the identification of regions with a high probability of disorder. All analyses were performed with the default parameters of the IUPred algorithm. The results presented here are based on the specific sequence input and the applied threshold for disorder classification.

### Computational analyses

#### Protein complex preparation and docking calculations:

The structure comprising HSP90β-HSP70(2)-HOP proteins was developed using the molecular comparative modeling technique, employing Modeller v10.4, the Modeller Python script^90^, and experimental template structures (PDB codes: 7KW7, 8EOB)^10,91^. The cryo-EM structure of human HSP90β (8EOB) served as the basis for obtaining coordinates for HSP90β (protomers A and B) in the developing model. To construct the assembly involving HSP70 and HOP, we utilized the sequences and atomic cryo-EM structure from the HSP90-HSP70-HOP-GR (7KW7) template. As these structures lacked certain residues, including those in the charged linker (Glu222 - Lys273), we incorporated them as intrinsic loops during computational processing. The target sequence for each HSP90β protomer was extracted from Uniprot ID: P08238. After model generation, we selected the optimal model based on the Discrete Optimized Protein Energy (DOPE) score. The final model included full-length HSP90 (excluding a ten-residue N-terminal disordered segment). For HOP and HSP70, we maintained the sequences provided in PDB:7KW7. The validated model, equipped with co-crystal ligands on each HSP90β protomer, was imported into Maestro v13.3 (Schrödinger LLC, 2022–3). Mutagenesis was performed to substitute Ser226/Ser255 with phosphomimetic conditions (Glu226/Glu255) and de-phosphorylated conditions (Ala226/Ala255) in both protomers of HSP90β. The preparation of all complexes utilized the Protein Preparation Wizard, a module for creating reliable, all-atom protein models. This involved restraining the assignment of bonds and bond orders, adding hydrogens, correcting formal charges, and filling missing side chains. Pre-processing steps included generating hetero states, H-bond assignment, and energy minimization using the Optimized Potentials for Liquid Simulations (OPLS3) force field, with a maximum root-mean-square deviation (RMSD) of 0.30 Å, employing the molecular mechanics engine Impact v9.6. Essential water atoms within 5 Å of the binding pocket were retained, while remaining waters were deleted. Structural refinement at neutral pH was carried out through the Epik v6.1 module^92^. The final refined structure served as the receptor for docking simulations. Ligands, such as ATP and ADP, underwent preparation with the LigPrep node, where the optimized ligand minimization algorithm yielded more conformers with numerous rotatable bonds, enhanced efficiency, and robustness. Different possible protonation states based on machine learning were generated, and ligand structures were minimized at pH values within the range of 7.0 and +/− 2.0, to guide the selection of protonation states on acidic/basic groups on ligands consistent with their pKa values, using the OPLS_3 force field, Premin, Truncated Newton Conjugate Gradient (TNCG), and Epik v6.1 nodes. Subsequently, a receptor grid was generated around the co-crystal ligand with default parameters. Docking experiments were executed on the nucleotide binding pockets of both protomers using the XP (extra-precision) Glide program (Glide v9.6) and Prime-MMGBSA (molecular mechanics generalized born surface area) modules, respectively. The best poses in the resulting docked complexes served as the initial complex structure for MD simulations^93^. *Molecular dynamics simulations*: The pentameric assemblies were prepared in the following combinations: 2xHSP90(Ser226Ser255)-2xHSP70-HOP, 2xHSP90(Glu226Glu255)-2xHSP70-HOP-, 2xHSP90(Ala226Ala255)-2xHSP70-HOP, each bound to either ATP or ADP. These complexes underwent individual 100 ns all-atomic molecular dynamics simulations using the Desmond v7.1 module of the MAESTRO Suite from Schrodinger (www.schrodinger.com). Before simulations, each assembly was built by embedding water molecules, adjusting temperature and pressure closer to the physiological environment through the OPLS3 force field and TIP4PEW water model. The system was neutralized with counter ions (Na^+^/Cl^−^) to balance the net charge in the simulation box. The particle mesh Ewald (PME) method^94^ was used for electrostatics with a 10 Å cut-off for Lennard-Jones interactions, and the SHAKE algorithm^95^ was applied to restrict the motion of all covalent bonds involving hydrogen atoms. The complex system underwent a six-step relaxation protocol before productive MD simulations. The solvated system was initially minimized with solute restraints and then without solute restraints, utilizing a hybrid method of steepest descent and the LBFGS (limited memory Broyden–Fletcher–Goldfarb–Shanno) algorithm^96,97^. The energy-minimized system underwent a brief 12 ps simulation within the NVT canonical ensemble at a temperature of 10 K, followed by a similar simulation in the isothermal-isobaric (NPT) ensemble at 10 K, with restraints on nonhydrogen solute atoms. Subsequently, the system was simulated for 24 ps in the NPT ensemble at 300 K with limited restraints on nonhydrogen solute atoms. In the final equilibration step, the system was simulated for 24 ps in the NPT ensemble at 300 K without constraints to reach an equilibrium state. The minimized and equilibrated system without restraints was then subjected to a 100 ns NPT simulation for production. The temperatures and pressures of the system in the initial simulations were controlled by Berendsen thermostats and barostats, respectively^96,97^. The relaxed system underwent productive simulations using the Nose’–Hoover thermostat at 300 K and the Martyna–Tobias–Klein barostat at 1.01325 bar pressure. Atomic-coordinate data for each receptor-ligand complex and system energies were recorded every 1000 ps. Residue-pair correlations were calculated along the MD trajectory using the script trj_essential_dynamics.py available in the Schrödinger suite. Additionally, the unexplored cryptic motions, distribution of secondary structural elements, and the array of protein folding in intrinsic disordered regions were thoroughly examined using the extracted meta-trajectory data from 1000 trajectories throughout the simulation period. The secondary structure elements (SSE) index was computed to illustrate the percentage occurrence of alpha-helices (α) and beta-strands (β) during the simulation period, delineated by residue.

### Immunoprecipitation of mCherry-HSP90

RFP Selector (NanoTag #N0410) resins were equilibrated with lysis buffer to prepare the resin. Cell lysates were then added and incubated with the resins at 4°C with head over tail rotation for 90 min. Following incubation, resins were washed twice with lysis buffer and once with PBS before elution with 2 × sample buffer and incubation at 95°C for 5 min. Eluents were then run on a 12.5% SDS-PAGE. For SILAC samples, heavy and light replicates were immunoprecipitated separately, before combined and separated by SDS gel electrophoresis.

### Chemical cross-linking

Cell lysates, with a concentration of approximately 3 μg μL^−1^, underwent cross-linking using disuccinimidyl suberate (DSS; ThermoFisher# 21655) at a concentration of 2.5 mM. This process occurred at room temperature for 1 h. To terminate the reaction, 0.8 M NH_4_OH (Sigma# 09859) was added, reaching a final concentration of 25 mM, and incubated at room temperature for an additional 15 min. The lysates were clarified through two rounds of centrifugation at 16,200× g for 15 min at 4°C before proceeding to separate HSP90 using immobilized PU-H71 or GA.

### SDS-PAGE and trypsin digestion

After elution from PU- or GA-beads, samples were loaded into 12.5% SDS-PAGE gel for separation. The entire lanes were cut into 10–15 bands and processed by in-gel digestion as described previously^19^. Briefly, gel bands were cut into small cubes, washed with 25 mM NH_4_HCO_3_/50% acetonitrile, reduced with 10 mM DTT (in 25 mM NH_4_HCO_3_) at 56°C for 1 h, alkylated with 55 mM iodoacetamide (in 25 mM NH_4_HCO_3_) in darkness for 45 min. Gel pieces were washed again with 25 mM NH_4_HCO_3_/50% acetonitrile and evaporated in a speed-vac to complete dryness. The dried gel samples were proteolyzed using varied volumes of trypsin (0.6–1.0 μg depending on the intensity of the gel bands) at 37°C for 4 h, before the extraction of tryptic peptides by 50% acetonitrile/2% acetic acid. Tryptic peptide mixture was concentrated down to ~7 μL before LC-MS/MS analysis. For validation experiments in Figure 3d,e, chemical precipitation and sample preparation for PTM analyses were performed as follows. For in-cell YK-B bait affinity purification, cells were plated in 10 cm plates at 6 × 10^6^ cells per plate and treated with 50 μM YK5-B for 4 h. Cells were next collected and lysed in 20 mM Tris pH 7.4, 150 mM NaCl and 1% NP40 buffer. Five hundred micrograms (500 μg) of total protein were incubated with streptavidin agarose beads (ThermoFisher Scientific) for 1 h and beads were washed with 20 mM Tris pH 7.4, 100 mM NaCl and 0.1% NP40 buffer (washing buffer). For in-lysate YK5-B bait affinity purification, cells were lysed in the above-mentioned lysis buffer. Streptavidin agarose beads were incubated with 50 μM YK5-biotin for 1 h, washed and added to 500 μg of total protein and incubated overnight. The beads were then washed with the washing buffer. For PU-H71 beads pull-down, 250 μg of the same protein lysates were incubated with 40 μl PU-H71 beads for 3 h and washed. The samples were applied onto SDS-PAGE. Resulting gels were washed 3 times in distilled deionized H_2_O for 15 min each and visualized by staining overnight with Simply Blue Coomassie stain (Thermo Fisher Scientific). Stained protein gel regions were typically excised into 6 gel sections per gel lane, and completely destained as described^19^. In-gel digestion was performed overnight with MS-grade trypsin (Trypsin Gold, Mass spectrometry grade, Promega) at 5 ng mL^−1^ in 50 mM NH_4_HCO_3_ digestion buffer and incubation at 37°C. After acidification with 10% formic acid (final concentration of 0.5–1% formic acid), peptides were extracted with 5% formic acid / 50% acetonitrile and resulting peptides were desalted using hand-packed, reversed phase Empore C18 Extraction Disks (3M, Cat#3M2215), following an established method^98^. Each of the 6 sections per sample, per gel lane, were excised and separately digested in-gel, at the same time, using the same batch and amount of trypsin. The peptides from each of these gel sections were purified and analyzed by nano-LC-MS/MS separately.

### LC-MS data acquisition, protein and phosphopeptide identification

Briefly, the digestion mixtures were injected into an Dionex Ultimate 3000 RSLCnano UHPLC system (Dionex Corporation, Sunnyvale, CA), and separated by a 75 μm × 25 cm PepMap RSLC column (100 Å, 2 μm) at a flow rate of ~450 nL min^−1^. The eluant was connected directly to a nanoelectrospray ionization source of an LTQ Orbitrap XL mass spectrometer (Thermo Scientific, Waltham, MA). LC-MS data were acquired in a data-dependent acquisition mode, cycling between a MS scan (m/z 315–2,000) acquired in the Orbitrap, followed by low-energy CID analysis on three most intense multiply charged precursors acquired in the linear ion trap. The centroided peak lists of the CID spectra were generated using PAVA searched against a database that is consisted of the Swiss-Prot protein database using Batch-Tag, a program of the University of California San Francisco Protein Prospector software, version 5.9.2. For identification of proteins in pull-down experiments, a precursor mass tolerance of 15 ppm and a fragment mass tolerance of 0.5 Da were used for protein database searches (trypsin as enzyme; 1 miscleavage; carbamidomethyl (C) as constant modification; acetyl (protein N-term), acetyl+oxidation (protein N-term), Met-loss (protein N-term), Met-loss+acetyl (protein N-term, oxidation (M)). Protein hits were reported with a Protein Prospector protein score ≥ 22, a protein discriminant score ≥ 0.0 and a peptide expectation value ≤ 0.01^99^. This set of thresholds of protein identification parameters does not return any substantial false positive protein hits from the randomized half of the concatenated database. After protein identification, PTM search was carried out with S/T/Y phosphorylation included in variable modifications among the identified proteins. A threshold of SLIP score >6 was imposed for false phosphorylation site assignment <5%^100^. Identified phosphopeptides were manually inspected by confirming the quality of MS/MS spectra and mass accuracy. Cross-linked peptides were identified using an integrated module in Protein Prospector, based on a bioinformation strategy developed in the UCSF Mass Spectrometry Facility^41,42,101,102^. Key cross-linked peptides were identified and confirmed by manually examining the returned spectrum, peptide scores, mass accuracy and absence from uncross-linked samples. For validation experiments in Figure 3e, MS data acquisition and processing were performed as follows. Desalted peptides were concentrated to a very small droplet by vacuum centrifugation and reconstituted in 10 mL 0.1% formic acid in H_2_O. Approximately 90% of the peptides were analyzed by nano-LC-MS/MS). A Q Exactive HF mass spectrometer was coupled directly to an EASY-nLC 1000 (Thermo Fisher Scientific) equipped with a self-packed 75 mm × 18 cm reverse phase column (ReproSil-Pur C18, 3M, Dr. Maisch GmbH, Germany) for peptide separation. Analytical column temperature was maintained at 50°C by a column oven (Sonation GmBH, Germany). Peptides were eluted with a 3–40% acetonitrile gradient over 60 min at a flow rate of 250 nL min^−1^. The mass spectrometer was operated in DDA mode with survey scans acquired at a resolution of 120,000 (at m/z 200) over a scan range of 300–1750 m/z. Up to 15 of the most abundant precursors from the survey scan were selected with an isolation window of 1.6 Th for fragmentation by higher-energy collisional dissociation with normalized collision energy (NCE) of 27. The maximum injection time for the survey and MS/MS scans was 20 ms and 60 ms, respectively; the ion target value (Automatic Gain Control) for survey and MS/MS scan modes was set to 3e^6^ and 1e^6^, respectively.

### Quantitation of phosphopeptides and crosslinked peptides

Manually-confirmed, high-confidence phosphopeptides and cross-linked peptides were quantified by the peak height of the extracted ion chromatogram of each peptide monoisotope mass. For phosphopeptide quantitation, the protein loading of HSP90 peptides in lysates or from pull-down experiments was normalize to a representative, isoform specific tryptic peptide, ELISNSSDALDK for HSP90α and ELISNASDALDK for HSP90β. Phosphopeptides with different charge state or miscleavages were considered as different measurements for quantitation of each phosphosite. To assess the relative phosphorylation levels of different phosphosites in cancer cells and non-transformed cells, the ion intensity values of all phosphopeptides for each phosphosite were summed. The average ion intensities of each phosphosite between cancer and non-transformed cells were compared. Cross-linked peptides were identified using an integrated module in Protein Prospector, based on a bioinformation strategy developed in the UCSF Mass Spectrometry Facility^41,42,101,102^. Key cross-linked peptides were identified and confirmed by manually examining the returned spectrum, peptide scores, mass accuracy and absence from uncross-linked samples. Cross-linked peptides identified from various samples were pooled together, and the cross-linking propensity of each cross-linked peptide was assessed by its cross-linking percentage^43^. Cross-linking percentage for each peptide pair was calculated using the following formula:

$$\%\text{XL}=\frac{\text{Cross}-\text{linked peptide Peak Height }(\text{PH})}{\sum \text{Cross}-\text{linked peptide PH}+\text{Dead}-\text{end XL }1\text{ PH}+\text{Dead}-\text{end XL }2\text{ PH}}$$

where the peak height is the apex peak height in LC-MS/MS runs. Dead-end XLs are cross-linker modified peptides where only one NHS-ester function of DSS is cross-linked to a Lys residue and the other NHS-ester function is hydrolyzed by water.

### Homology modeling

The mouse HSP90 sequences for both alpha and beta isoforms were aligned and the models were built using an open conformation template (PDB: 2IOQ), a closed conformation template (PDB: 2CG9), and an HSP70-bound model (derived from a cryo-EM structure of HSP90•HSP70•GR complex^10^ using UCSF Modeller. Structural visualization and analysis were carried out using UCSF Chimera.

### Statistics and reproducibility

Unless as specified above under Protein identification and Bioinformatics analyses, statistics were performed, and graphs were generated, using Prism 10 software (GraphPad). Statistical significance was determined using Student’s t-Tests or ANOVA, as indicated. Means and standard errors were reported for all results unless otherwise specified. Effects achieving 95% confidence interval (i.e., *p* < 0.05) were interpreted as statistically significant. No statistical methods were used to pre-determine sample sizes, but these are similar to those generally employed in the field. No samples were excluded from any analysis unless explicitly stated.

#### Reporting summary.

Further information on research design is available in the Nature Research Reporting Summary linked to this article.

## Data Availability

The source data underlying all main and supplementary figures are provided with this paper as a Source Data file. Datasets and analytics associated with epichaperomics and proteomics analyses are available in the Supplementary Information as Supplementary Data 1 through 6. LC-MS data (i.e., proteomics and epichaperomics raw mass spectrometry data, peak lists, and results) that support the findings of this study are deposited to the ProteomeXchange Consortium via the PRIDE partner repository with the dataset identifier PXD050251 [**Reviewer account details**: Username: reviewer_pxd050251@ebi.ac.uk; Password: TmZMDQ0W]. Protein sequences (FASTA files) were obtained from UniProt (https://www.uniprot.org/). MD simulations data were deposited in Zenodo [https://doi.org/10.5281/zenodo.10800912]^103^. Source data are provided with this paper.

## References

[1] Bludau I. & Aebersold R. Proteomic and interactomic insights into the molecular basis of cell functional diversity. Nat. Rev. Mol. Cell Biol. 21, 327–340 (2020). 32235894 10.1038/s41580-020-0231-2

[2] Nussinov R., Tsai C. J. & Jang H. Protein ensembles link genotype to phenotype. PLoS Comput. Biol. 15, e1006648 (2019). 31220071 10.1371/journal.pcbi.1006648 PMC6586255

[3] Chiosis G., Digwal C. S., Trepel J. B. & Neckers L. Structural and functional complexity of HSP90 in cellular homeostasis and disease. Nat. Rev. Mol. Cell Biol. 24, 797–815 (2023). 37524848 10.1038/s41580-023-00640-9 PMC10592246

[4] Schopf F. H., Biebl M. M. & Buchner J. The HSP90 chaperone machinery. Nat. Rev. Mol. Cell Biol. 18, 345–360 (2017). 28429788 10.1038/nrm.2017.20

[5] Krukenberg K. A., Street T. O., Lavery L. A. & Agard D. A. Conformational dynamics of the molecular chaperone Hsp90. Q. Rev. Biophys. 44, 229–255 (2011). 21414251 10.1017/S0033583510000314 PMC5070531

[6] Biebl M. M. & Buchner J. Structure, Function, and Regulation of the Hsp90 Machinery. Cold Spring Harb. Perspect. Biol. 11, (2019). 10.1101/cshperspect.a034017 PMC6719599 30745292

[7] Street T. O., Lavery L. A., Verba K. A., Lee C. T., Mayer M. P. & Agard D. A. Cross-monomer substrate contacts reposition the Hsp90 N-terminal domain and prime the chaperone activity. J. Mol. Biol. 415, 3–15 (2012). 22063096 10.1016/j.jmb.2011.10.038 PMC3282117

[8] Street T. O., Lavery L. A. & Agard D. A. Substrate binding drives large-scale conformational changes in the Hsp90 molecular chaperone. Mol. Cell 42, 96–105 (2011). 21474071 10.1016/j.molcel.2011.01.029 PMC3105473

[9] Street T. O. Elucidating the mechanism of substrate recognition by the bacterial Hsp90 molecular chaperone. J. Mol. Biol. 426, 2393–2404 (2014). 24726919 10.1016/j.jmb.2014.04.001 PMC5322795

[10] Wang R. Y., Noddings C. M., Kirschke E., Myasnikov A. G., Johnson J. L. & Agard D. A. Structure of Hsp90-Hsp70-Hop-GR reveals the Hsp90 client-loading mechanism. Nature 601, 460–464 (2022). 34937942 10.1038/s41586-021-04252-1 PMC9179170

[11] Dean M. E. & Johnson J. L. Human Hsp90 cochaperones: perspectives on tissue-specific expression and identification of cochaperones with similar in vivo functions. Cell Stress Chaperones 26, 3–13 (2021). 33037995 10.1007/s12192-020-01167-0 PMC7736379

[12] Backe S. J., Sager R. A., Woodford M. R., Makedon A. M. & Mollapour M. Post-translational modifications of Hsp90 and translating the chaperone code. J. Biol. Chem. 295, 11099–11117 (2020). 32527727 10.1074/jbc.REV120.011833 PMC7415980

[13] Rodina A. The epichaperome is an integrated chaperome network that facilitates tumour survival. Nature 538, 397–401 (2016). 27706135 10.1038/nature19807 PMC5283383

[14] Svirsky S. E., Li Y., Henchir J., Rodina A., Carlson S. W., Chiosis G. & Dixon C. E. Experimental traumatic brain injury increases epichaperome formation. Neurobiol. Dis. 188, 106331 (2023). 37863370 10.1016/j.nbd.2023.106331 PMC10698712

[15] Inda M. C. The epichaperome is a mediator of toxic hippocampal stress and leads to protein connectivity-based dysfunction. Nat. Commun. 11, 319 (2020). 31949159 10.1038/s41467-019-14082-5 PMC6965647

[16] Ginsberg S. D., Joshi S., Sharma S., Guzman G., Wang T., Arancio O. & Chiosis G. The penalty of stress - Epichaperomes negatively reshaping the brain in neurodegenerative disorders. J. Neurochem. 159, 958–979 (2021). 34657288 10.1111/jnc.15525 PMC8688321

[17] Joshi S., Wang T., Araujo T. L. S., Sharma S., Brodsky J. L. & Chiosis G. Adapting to stress - chaperome networks in cancer. Nat. Rev. Cancer 18, 562–575 (2018). 29795326 10.1038/s41568-018-0020-9 PMC6108944

[18] Ginsberg S. D., Sharma S., Norton L. & Chiosis G. Targeting stressor-induced dysfunctions in protein-protein interaction networks via epichaperomes. Trends Pharmacol. Sci. 44, 20–33 (2023). 36414432 10.1016/j.tips.2022.10.006 PMC9789192

[19] Rodina A. Systems-level analyses of protein-protein interaction network dysfunctions via epichaperomics identify cancer-specific mechanisms of stress adaptation. Nat. Commun. 14, 3742 (2023). 37353488 10.1038/s41467-023-39241-7 PMC10290137

[20] Kishinevsky S. HSP90-incorporating chaperome networks as biosensor for disease-related pathways in patient-specific midbrain dopamine neurons. Nat. Commun. 9, 4345 (2018). 30341316 10.1038/s41467-018-06486-6 PMC6195591

[21] Zong H. A Hyperactive Signalosome in Acute Myeloid Leukemia Drives Addiction to a Tumor-Specific Hsp90 Species. Cell Rep. 13, 2159–2173 (2015). 26628369 10.1016/j.celrep.2015.10.073 PMC4699804

[22] Sugita M. Targeting the epichaperome as an effective precision medicine approach in a novel PML-SYK fusion acute myeloid leukemia. NPJ Precis. Oncol. 5, 44 (2021). 34040147 10.1038/s41698-021-00183-2 PMC8155064

[23] Bolaender A. Chemical tools for epichaperome-mediated interactome dysfunctions of the central nervous system. Nat. Commun. 12, 4669 (2021). 34344873 10.1038/s41467-021-24821-2 PMC8333062

[24] Araki R. Crucial role of c-Myc in the generation of induced pluripotent stem cells. Stem Cells 29, 1362–1370 (2011). 21732496 10.1002/stem.685

[25] Varlakhanova N. V. myc maintains embryonic stem cell pluripotency and self-renewal. Differentiation 80, 9–19 (2010). 20537458 10.1016/j.diff.2010.05.001 PMC2916696

[26] Wang J. c-Myc is required for maintenance of glioma cancer stem cells. PLoS One 3, e3769 (2008). 19020659 10.1371/journal.pone.0003769 PMC2582454

[27] Kourtis N. Oncogenic hijacking of the stress response machinery in T cell acute lymphoblastic leukemia. Nat. Med. 24, 1157–1166 (2018). 30038221 10.1038/s41591-018-0105-8 PMC6082694

[28] Sharma S. Unraveling the Mechanism of Epichaperome Modulation by Zelavespib: Biochemical Insights on Target Occupancy and Extended Residence Time at the Site of Action. Biomedicines 11, (2023). 10.3390/biomedicines11102599 PMC10604720 37892973

[29] Minami Y., Kawasaki H., Miyata Y., Suzuki K. & Yahara I. Analysis of native forms and isoform compositions of the mouse 90-kDa heat shock protein, HSP90. J. Biol. Chem. 266, 10099–10103 (1991). 2037568

[30] Pillarsetty N. Paradigms for Precision Medicine in Epichaperome Cancer Therapy. Cancer Cell 36, 559–573.e557 (2019). 31668946 10.1016/j.ccell.2019.09.007 PMC6996250

[31] Niwa H., Miyazaki J. & Smith A. G. Quantitative expression of Oct-3/4 defines differentiation, dedifferentiation or self-renewal of ES cells. Nat. Genet. 24, 372–376 (2000). 10742100 10.1038/74199

[32] Lee J., Go Y., Kang I., Han Y. M. & Kim J. Oct-4 controls cell-cycle progression of embryonic stem cells. Biochem. J. 426, 171–181 (2010). 19968627 10.1042/BJ20091439 PMC2825734

[33] Kurosawa H. Methods for inducing embryoid body formation: in vitro differentiation system of embryonic stem cells. J Biosci Bioeng 103, 389–398 (2007). 17609152 10.1263/jbb.103.389

[34] Miller J. D. Human iPSC-based modeling of late-onset disease via progerin-induced aging. Cell Stem Cell 13, 691–705 (2013). 24315443 10.1016/j.stem.2013.11.006 PMC4153390

[35] Moulick K. Affinity-based proteomics reveal cancer-specific networks coordinated by Hsp90. Nat. Chem. Biol. 7, 818–826 (2011). 21946277 10.1038/nchembio.670 PMC3265389

[36] Chen P. B. Hdac6 regulates Tip60-p400 function in stem cells. Elife 2, e01557 (2013). 24302573 10.7554/eLife.01557 PMC3843111

[37] Brickner D. G. Transcription factor binding to a DNA zip code controls interchromosomal clustering at the nuclear periphery. Dev. Cell 22, 1234–1246 (2012). 22579222 10.1016/j.devcel.2012.03.012 PMC3376219

[38] Beebe K. Posttranslational modification and conformational state of heat shock protein 90 differentially affect binding of chemically diverse small molecule inhibitors. Oncotarget 4, 1065–1074 (2013). 23867252 10.18632/oncotarget.1099 PMC3759666

[39] Maiti S. & Picard D. Cytosolic Hsp90 Isoform-Specific Functions and Clinical Significance. Biomolecules 12, (2022). 10.3390/biom12091166 PMC9496497 36139005

[40] Chu F. Unraveling the interface of signal recognition particle and its receptor by using chemical cross-linking and tandem mass spectrometry. Proc. Natl. Acad. Sci. U. S. A. 101, 16454–16459 (2004). 15546976 10.1073/pnas.0407456101 PMC528904

[41] Karagoz G. E., Acosta-Alvear D., Nguyen H. T., Lee C. P., Chu F. & Walter P. An unfolded protein-induced conformational switch activates mammalian IRE1. Elife 6, (2017). 10.7554/eLife.30700 PMC5699868 28971800

[42] Chu F., Thornton D. T. & Nguyen H. T. Chemical cross-linking in the structural analysis of protein assemblies. Methods 144, 53–63 (2018). 29857191 10.1016/j.ymeth.2018.05.023 PMC6051896

[43] Chu F., Hogan D., Gupta R., Gao X. Z., Nguyen H. T. & Cote R. H. Allosteric Regulation of Rod Photoreceptor Phosphodiesterase 6 (PDE6) Elucidated by Chemical Cross-Linking and Quantitative Mass Spectrometry. J. Mol. Biol. 431, 3677–3689 (2019). 31394113 10.1016/j.jmb.2019.07.035 PMC6733632

[44] Stebbins C. E., Russo A. A., Schneider C., Rosen N., Hartl F. U. & Pavletich N. P. Crystal structure of an Hsp90-geldanamycin complex: targeting of a protein chaperone by an antitumor agent. Cell 89, 239–250 (1997). 9108479 10.1016/s0092-8674(00)80203-2

[45] Huck J. D., Que N. L. S., Sharma S., Taldone T., Chiosis G. & Gewirth D. T. Structures of Hsp90alpha and Hsp90beta bound to a purine-scaffold inhibitor reveal an exploitable residue for drug selectivity. Proteins 87, 869–877 (2019). 31141217 10.1002/prot.25750 PMC6718336

[46] Immormino R. M., Kang Y., Chiosis G. & Gewirth D. T. Structural and quantum chemical studies of 8-aryl-sulfanyl adenine class Hsp90 inhibitors. J. Med. Chem. 49, 4953–4960 (2006). 16884307 10.1021/jm060297x

[47] Jeong H., Kang B. H. & Lee C. Crystallization and preliminary X-ray diffraction analysis of Trap1 complexed with Hsp90 inhibitors. Acta Crystallogr F Struct Biol Commun 70, 1683–1687 (2014). 25484226 10.1107/S2053230X14024959 PMC4259240

[48] Prodromou C., Roe S. M., O’Brien R., Ladbury J. E., Piper P. W. & Pearl L. H. Identification and structural characterization of the ATP/ADP-binding site in the Hsp90 molecular chaperone. Cell 90, 65–75 (1997). 9230303 10.1016/s0092-8674(00)80314-1

[49] Ali M. M. Crystal structure of an Hsp90-nucleotide-p23/Sba1 closed chaperone complex. Nature 440, 1013–1017 (2006). 16625188 10.1038/nature04716 PMC5703407

[50] Castelli M. Molecular mechanisms of chaperone-directed protein folding: Insights from atomistic simulations. Protein Sci. 33, e4880 (2023). 38145386 10.1002/pro.4880 PMC10895457

[51] Yan P. Molecular Stressors Engender Protein Connectivity Dysfunction through Aberrant N-Glycosylation of a Chaperone. Cell Rep. 31, 107840 (2020). 32610141 10.1016/j.celrep.2020.107840 PMC7372946

[52] Wang X. & Huang L. Identifying dynamic interactors of protein complexes by quantitative mass spectrometry. Mol. Cell. Proteomics 7, 46–57 (2008). 17934176 10.1074/mcp.M700261-MCP200

[53] Weidenauer L. & Quadroni M. Phosphorylation in the Charged Linker Modulates Interactions and Secretion of Hsp90beta. Cells 10, (2021). 10.3390/cells10071701 PMC8304327 34359868

[54] Schlecht R., Erbse A. H., Bukau B. & Mayer M. P. Mechanics of Hsp70 chaperones enables differential interaction with client proteins. Nat. Struct. Mol. Biol. 18, 345–351 (2011). 21278757 10.1038/nsmb.2006

[55] Dhanasekaran R., Deutzmann A., Mahauad-Fernandez W. D., Hansen A. S., Gouw A. M. & Felsher D. W. The MYC oncogene - the grand orchestrator of cancer growth and immune evasion. Nat. Rev. Clin. Oncol. 19, 23–36 (2022). 34508258 10.1038/s41571-021-00549-2 PMC9083341

[56] Chen G., Yin S., Zeng H., Li H. & Wan X. Regulation of Embryonic Stem Cell Self-Renewal. Life (Basel) 12, (2022). 10.3390/life12081151 PMC9410528 36013330

[57] Mohiuddin I. S., Wei S. J. & Kang M. H. Role of OCT4 in cancer stem-like cells and chemotherapy resistance. Biochim Biophys Acta Mol Basis Dis 1866, 165432 (2020). 30904611 10.1016/j.bbadis.2019.03.005 PMC6754810

[58] Joshi S. Pharmacologically controlling protein-protein interactions through epichaperomes for therapeutic vulnerability in cancer. Commun. Biol. 4, 1333 (2021). 34824367 10.1038/s42003-021-02842-3 PMC8617294

[59] Carter B. Z. Epichaperome inhibition targets TP53-mutant AML and AML stem/progenitor cells. Blood 142, 1056–1070 (2023). 37339579 10.1182/blood.2022019047 PMC10656725

[60] Yang J. Guidelines and definitions for research on epithelial-mesenchymal transition. Nat. Rev. Mol. Cell Biol. 21, 341–352 (2020). 32300252 10.1038/s41580-020-0237-9 PMC7250738

[61] Scroggins B. T. An acetylation site in the middle domain of Hsp90 regulates chaperone function. Mol. Cell 25, 151–159 (2007). 17218278 10.1016/j.molcel.2006.12.008 PMC1839984

[62] Soroka J., Wandinger S. K., Mausbacher N., Schreiber T., Richter K., Daub H. & Buchner J. Conformational switching of the molecular chaperone Hsp90 via regulated phosphorylation. Mol. Cell 45, 517–528 (2012). 22365831 10.1016/j.molcel.2011.12.031

[63] Mollapour M. Swe1Wee1-dependent tyrosine phosphorylation of Hsp90 regulates distinct facets of chaperone function. Mol. Cell 37, 333–343 (2010). 20159553 10.1016/j.molcel.2010.01.005 PMC2824606

[64] Mollapour M. Asymmetric Hsp90 N domain SUMOylation recruits Aha1 and ATP-competitive inhibitors. Mol. Cell 53, 317–329 (2014). 24462205 10.1016/j.molcel.2013.12.007 PMC3964875

[65] Xu W. Hsp90 middle domain phosphorylation initiates a complex conformational program to recruit the ATPase-stimulating cochaperone Aha1. Nat. Commun. 10, 2574 (2019). 31189925 10.1038/s41467-019-10463-y PMC6561935

[66] Retzlaff M., Stahl M., Eberl H. C., Lagleder S., Beck J., Kessler H. & Buchner J. Hsp90 is regulated by a switch point in the C-terminal domain. EMBO Rep 10, 1147–1153 (2009). 19696785 10.1038/embor.2009.153 PMC2759728

[67] Rehn A. A methylated lysine is a switch point for conformational communication in the chaperone Hsp90. Nat. Commun. 11, 1219 (2020). 32139682 10.1038/s41467-020-15048-8 PMC7057950

[68] Kundrat L. & Regan L. Identification of residues on Hsp70 and Hsp90 ubiquitinated by the cochaperone CHIP. J. Mol. Biol. 395, 587–594 (2010). 19913553 10.1016/j.jmb.2009.11.017 PMC2917188

[69] Jahn M. The charged linker of the molecular chaperone Hsp90 modulates domain contacts and biological function. Proc. Natl. Acad. Sci. U. S. A. 111, 17881–17886 (2014). 25468961 10.1073/pnas.1414073111 PMC4273377

[70] Lees-Miller S. P. & Anderson C. W. Two human 90-kDa heat shock proteins are phosphorylated in vivo at conserved serines that are phosphorylated in vitro by casein kinase II. J. Biol. Chem. 264, 2431–2437 (1989). 2492519

[71] Kim S. W. Casein Kinase 2 (CK2)-mediated Phosphorylation of Hsp90β as a Novel Mechanism of Rifampin-induced MDR1 Expression. J. Biol. Chem. 290, 17029–17040 (2015). 25995454 10.1074/jbc.M114.624106 PMC4505446

[72] Zakharia K., Miyabe K., Wang Y., Wu D., Moser C. D., Borad M. J. & Roberts L. R. Preclinical In Vitro and In Vivo Evidence of an Antitumor Effect of CX-4945, a Casein Kinase II Inhibitor, in Cholangiocarcinoma. Transl. Oncol. 12, 143–153 (2019). 30316146 10.1016/j.tranon.2018.09.005 PMC6187100

[73] St-Denis N. A. & Litchfield D. W. Protein kinase CK2 in health and disease: From birth to death: the role of protein kinase CK2 in the regulation of cell proliferation and survival. Cell. Mol. Life Sci. 66, 1817–1829 (2009). 19387552 10.1007/s00018-009-9150-2 PMC11115660

[74] Buchou T. Disruption of the regulatory beta subunit of protein kinase CK2 in mice leads to a cell-autonomous defect and early embryonic lethality. Mol Cell Biol 23, 908–915 (2003). 12529396 10.1128/MCB.23.3.908-915.2003 PMC140710

[75] Ruzzene M. & Pinna L. A. Addiction to protein kinase CK2: a common denominator of diverse cancer cells? Biochim Biophys Acta 1804, 499–504 (2010). 19665589 10.1016/j.bbapap.2009.07.018

[76] Borgo C. Generation and quantitative proteomics analysis of CK2alpha/alpha’((−/−)) cells. Sci. Rep. 7, 42409 (2017). 28209983 10.1038/srep42409 PMC5314375

[77] Hanahan D. Hallmarks of Cancer: New Dimensions. Cancer Discov. 12, 31–46 (2022). 35022204 10.1158/2159-8290.CD-21-1059

[78] Labusca L. & Mashayekhi K. Human adult pluripotency: Facts and questions. World J. Stem Cells 11, 1–12 (2019). 30705711 10.4252/wjsc.v11.i1.1 PMC6354101

[79] Lee L. J., Papadopoli D., Jewer M., Del Rincon S., Topisirovic I., Lawrence M. G. & Postovit L. M. Cancer Plasticity: The Role of mRNA Translation. Trends Cancer 7, 134–145 (2021). 33067172 10.1016/j.trecan.2020.09.005 PMC8023421

[80] Rodina A. Identification of an allosteric pocket on human hsp70 reveals a mode of inhibition of this therapeutically important protein. Chem. Biol. 20, 1469–1480 (2013). 24239008 10.1016/j.chembiol.2013.10.008 PMC3985611

[81] Kang Y. Heat shock protein 70 inhibitors. 1. 2,5’-thiodipyrimidine and 5-(phenylthio)pyrimidine acrylamides as irreversible binders to an allosteric site on heat shock protein 70. J. Med. Chem. 57, 1188–1207 (2014). 24548207 10.1021/jm401551n PMC3983365

[82] Rodina A. Affinity purification probes of potential use to investigate the endogenous Hsp70 interactome in cancer. ACS Chem. Biol. 9, 1698–1705 (2014). 24934503 10.1021/cb500256u PMC4134716

[83] Taldone T. Heat shock protein 70 inhibitors. 2. 2,5’-thiodipyrimidines, 5-(phenylthio)pyrimidines, 2-(pyridin-3-ylthio)pyrimidines, and 3-(phenylthio)pyridines as reversible binders to an allosteric site on heat shock protein 70. J. Med. Chem. 57, 1208–1224 (2014). 24548239 10.1021/jm401552y PMC3983364

[84] Shrestha L., Patel H. J. & Chiosis G. Chemical Tools to Investigate Mechanisms Associated with HSP90 and HSP70 in Disease. Cell Chem. Biol. 23, 158–172 (2016). 26933742 10.1016/j.chembiol.2015.12.006 PMC4779498

[85] Taldone T. Design, synthesis, and evaluation of small molecule Hsp90 probes. Bioorg. Med. Chem. 19, 2603–2614 (2011). 21459002 10.1016/j.bmc.2011.03.013 PMC3143825

[86] Guasparri I., Bubman D. & Cesarman E. EBV LMP2A affects LMP1-mediated NF-kappaB signaling and survival of lymphoma cells by regulating TRAF2 expression. Blood 111, 3813–3820 (2008). 18230756 10.1182/blood-2007-03-080309 PMC2275034

[87] Hooper M., Hardy K., Handyside A., Hunter S. & Monk M. HPRT-deficient (Lesch-Nyhan) mouse embryos derived from germline colonization by cultured cells. Nature 326, 292–295 (1987). 3821905 10.1038/326292a0

[88] Roychowdhury T., Santhaseela A. R., Sharma S., Panchal P., Rodina A. & Chiosis G. Use of Native-PAGE for the Identification of Epichaperomes in Cell Lines. Methods Mol. Biol. 2693, 175–191 (2023). 37540435 10.1007/978-1-0716-3342-7_14 PMC10448758

[89] Corben A. D. Ex vivo treatment response of primary tumors and/or associated metastases for preclinical and clinical development of therapeutics. J. Vis. Exp., e52157 (2014). 25350385 10.3791/52157 PMC4315621

[90] Sali A. & Blundell T. L. Comparative protein modelling by satisfaction of spatial restraints. J. Mol. Biol. 234, 779–815 (1993). 8254673 10.1006/jmbi.1993.1626

[91] Srivastava D., Yadav R. P., Singh S., Boyd K. & Artemyev N. O. Unique interface and dynamics of the complex of HSP90 with a specialized cochaperone AIPL1. Structure 31, 309–317 e305 (2023). 36657440 10.1016/j.str.2022.12.014 PMC9992320

[92] Johnston R. C. Epik: pK(a) and Protonation State Prediction through Machine Learning. J. Chem. Theory Comput. 19, 2380–2388 (2023). 37023332 10.1021/acs.jctc.3c00044

[93] Castelli M. How aberrant N-glycosylation can alter protein functionality and ligand binding: An atomistic view. Structure 31, 987–1004.e1008 (2023). 37343552 10.1016/j.str.2023.05.017 PMC10526633

[94] Darden T., York D. & Pedersen L. Particle Mesh Ewald - an N.Log(N) Method for Ewald Sums in Large Systems. J. Chem. Phys. 98, 10089–10092 (1993).

[95] Ryckaert J., Ciccotti G. & Berendsen H. J. C. Numerical integration of the cartesian equations of motion of a system with constraints: molecular dynamics of n-alkanes. J. Comput. Phys. 23, 327–341 (1977).

[96] Hayes J. M. Kinetics, in silico docking, molecular dynamics, and MM-GBSA binding studies on prototype indirubins, KT5720, and staurosporine as phosphorylase kinase ATP-binding site inhibitors: the role of water molecules examined. Proteins 79, 703–719 (2011). 21287607 10.1002/prot.22890

[97] Shan Y., Kim E. T., Eastwood M. P., Dror R. O., Seeliger M. A. & Shaw D. E. How does a drug molecule find its target binding site? J. Am. Chem. Soc. 133, 9181–9183 (2011). 21545110 10.1021/ja202726y PMC3221467

[98] Rappsilber J., Mann M. & Ishihama Y. Protocol for micro-purification, enrichment, pre-fractionation and storage of peptides for proteomics using StageTips. Nat. Protoc. 2, 1896–1906 (2007). 17703201 10.1038/nprot.2007.261

[99] Wu T., Nance J., Chu F. & Fazzio T. G. Characterization of R-Loop-Interacting Proteins in Embryonic Stem Cells Reveals Roles in rRNA Processing and Gene Expression. Mol. Cell. Proteomics 20, 100142 (2021). 34478875 10.1016/j.mcpro.2021.100142 PMC8461376

[100] Zhou Y., Qiu L., Sterpka A., Wang H., Chu F. & Chen X. Comparative Phosphoproteomic Profiling of Type III Adenylyl Cyclase Knockout and Control, Male, and Female Mice. Front. Cell. Neurosci. 13, 34 (2019). 30814930 10.3389/fncel.2019.00034 PMC6381875

[101] Chu F., Baker P. R., Burlingame A. L. & Chalkley R. J. Finding chimeras: a bioinformatics strategy for identification of cross-linked peptides. Mol. Cell. Proteomics 9, 25–31 (2010). 19809093 10.1074/mcp.M800555-MCP200 PMC2808265

[102] Zeng-Elmore X. Molecular architecture of photoreceptor phosphodiesterase elucidated by chemical cross-linking and integrative modeling. J. Mol. Biol. 426, 3713–3728 (2014). 25149264 10.1016/j.jmb.2014.07.033 PMC4253074

[103] Pasala C., Digwal C. S., Sharma S. & Chiosis G. Molecular dynamics simulation data associated with manuscript: Phosphorylation-Driven Epichaperome Assembly: A Critical Regulator of Cellular Adaptability and Proliferation [Data set]. Zenodo, 10.5281/zenodo.10800912 (2024). PMC11484706 39414766

[104] Kirschke E., Goswami D., Southworth D., Griffin P. R. & Agard D. A. Glucocorticoid receptor function regulated by coordinated action of the Hsp90 and Hsp70 chaperone cycles. Cell 157, 1685–1697 (2014). 24949977 10.1016/j.cell.2014.04.038 PMC4087167

